# Hydrophobic Surface Modification Enables Tandem Ag/Cu Catalysis for CO_2_ Electroreduction

**DOI:** 10.1021/acsami.5c22445

**Published:** 2026-04-20

**Authors:** Yu-Cheng Liu, Kang-Shun Peng, Yu-Jhih Shen, Shao-Hui Hsu, Ching-Hsuan Chou, Ya-Ching Chang, Ming-Hsuan Li, Ying-Rui Lu, Sung-Fu Hung

**Affiliations:** † Department of Applied Chemistry and Center for Emergent Functional Matter Science, 34914 National Yang Ming Chiao Tung University, Hsinchu 300, Taiwan; ‡ Taiwan Semiconductor Research Institute, 63353 National Institutes of Applied Research, Hsinchu 300, Taiwan; § 57815 National Synchrotron Radiation Research Center, Hsinchu 300, Taiwan; ∥ Department of Medicinal and Applied Chemistry, Kaohsiung Medical University, Kaohsiung 807378, Taiwan

**Keywords:** CO_2_RR, Flow Cell, Tandem Catalysts, Hydrophobic Modification, In-Situ Raman

## Abstract

Ag–Cu tandem catalysts are a promising route to boost C_2+_ formation during CO_2_ electroreduction; however, well-defined layered Ag/Cu catalysts fabricated by PVD/sputtering without an ionomer behave like pure Cu in flow cells, showing no tandem enhancement. Contact-angle measurements indicate that the exposed Ag surface lowers overall hydrophobicity, restricting CO_2_ transport to Ag and suppressing tandem pathways. To address this limitation, in this study, we adopt a hydrophobic surface modification using 1-dodecanethiol (DDT). The resulting DDT–Ag/Cu achieves 74.09 ± 1.69% Faradaic efficiency toward C_2+_ products with a partial current density of 370.5 ± 8.45 mA cm^–2^ at 500 mA cm^–2^, outperforming benchmark Cu and unmodified Ag/Cu under optimized conditions (by ∼65%). DDT–Ag/Cu also enhances ethanol selectivity, increasing the ethanol-to-ethylene ratio from ∼0.5 to ∼1.0. In situ Raman spectroscopy reveals distinct intermediates under hydrophobic conditions. These results clarify the intrinsic behavior of Ag–Cu tandem catalysis and offer a practical strategy to boost tandem performance in flow-cell CO_2_ electroreduction.

## Graphical abstract

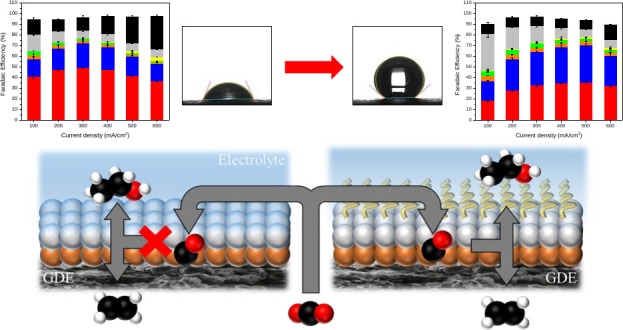

## 1 Introduction

Electrocatalytic CO_2_ reduction reaction (CO_2_RR) is widely regarded as an effective approach to mitigate atmospheric CO_2_ while generating value-added products.^1–3^ To enable practical application of CO_2_RR, numerous high-efficient catalysts have been developed to access diverse products,^4–6^ and it is now clear the local reaction environments strongly modulate activity and selectivity.^7,8^ Reactor configuration, in particular, plays a decisive role in shaping these environments and determining catalytic behavior. Compared with conventional H-type cells—where performance is constrained by the limited solubility of CO_2_ in aqueous electrolytes—flow cells provide more efficient gas delivery and support higher current densities.^9,10^ In a flow-cell configuration, CO_2_ is supplied from the gas chamber while electrolyte permeates the catalytic surface first, establishing a gas–liquid–solid triple-phase interface that governs mass transport, intermediate diffusion, and overall reaction selectivity.^11,12^

Among various catalysts, Copper, as the primary catalyst, has unique advantages in converting CO_2_ into multicarbon (C_2__**+**_) products.^13,14^ Extensive efforts have targeted improving the selectivity and activity of Cu-based catalysts toward C_2__**+**_ products, including alloying, interface engineering, and oxidation state control.^15–22^ Among these strategies, tandem catalysis—coupling Cu with CO-producing catalysts such as Ag, Au, or single atom catalysts—has emerged as a particularly effective route to enhance C–C coupling.^23–25^ For instance, Chen et al. established a Cu–Ag tandem platform on a GDE and reported that introducing Ag increased the **C**_**2+**_ partial current density from 37 to 160 mA cm^–2^ at −0.70 V vs RHE in 1 M KOH, highlighting the benefit of tandem coupling but still at a moderate **C**_**2+**_ production rate compared to later high-rate systems.^26^ Meanwhile, Au-containing Cu systems have also been investigated for oxygenate pathways; for example, an atomically dispersed Cu–Au alloy achieved an acetate Faradaic efficiency of ∼39% with a partial current density of 217 mA cm^–2^ in an alkaline flow cell, indicating that Au–Cu interfaces can steer selectivity but the overall partial current density remains below high-rate **C**_**2+**_ benchmarks.^27^ These precedents motivate strategies that simultaneously maintain gas-transport pathways and stabilize effective interfacial configurations to unlock stronger tandem performance at industrially relevant current densities. Notably, Ag–Cu assemblies have repeatedly demonstrated strong synergistic behavior, promoting CO_2_-to-C_**2+**_ conversion via CO spillover.^28^

The tandem concept relies on locally enriching CO*—the key intermediate for C_2+_ formation—to enhance C–C coupling on Cu. In most reports, Ag–Cu systems are paired with additional design strategies (e.g., controlled crystal phases, single-atom alloys, or engineered architectures) to further improve performance.^29–33^

Interfaces are increasingly recognized as a key design lever in electrocatalysis, as they regulate not only charge transfer and electronic structure but also mass transport, wettability, and selectivity under practical current densities. For instance, Chen et al. showed that interfacial passivation engineering can simultaneously sustain and enhance the performance of acidic OER electrodes, while Tian et al. demonstrated that ionic liquid–TiO_2_–CuO_*x*_ composite interfaces coupled with directional gas transport markedly improve methane-to-ethanol conversion efficiency. Moreover, Sassenburg et al. highlighted that electrode interfaces can dynamically restructure during high-rate operation, leading to changes in active-phase exposure and interfacial connectivity that directly impact selectivity and durability in tandem systems.^34–36^ In these context, hydrophobic microenvironment engineering has emerged as an effective strategy to sustain high-rate CO_2_ electrolysis in GDE-based tandem architectures, where interfacial wettability critically governs CO_2_ accessibility, gas transport, and resistance to flooding. Mainstream approaches—such as fluorinated surface coatings and polymer/ionomer-based encapsulation—can enhance hydrophobicity and stabilize gas pathways. However, these modifications may also introduce trade-offs, including increased mass-transport resistance, partial masking of active sites, perturbation of the local ionic environment, and, in some cases, altered product selectivity arising from direct polymer–catalyst interactions.^37^ These effects complicate efforts to unambiguously isolate the intrinsic tandem interaction between metallic Ag and Cu.^38,39^

Herein, we construct an ionomer-free, well-defined Ag/Cu model *tandem* catalyst by sequentially sputtering Cu and thermal evaporation of Ag onto carbon paper, enabling direct evaluation of intrinsic *tandem* behavior in a flow cell configuration. Unexpectedly, the Ag/Cu electrode exhibits comparably to that of Cu-only electrodes. Contact angle measurements reveal that the Ag overlayer is sufficiently hydrophilic to become electrolyte-wetted, thereby disrupting CO_2_ diffusion to Ag sites and suppressing tandem pathways. Motivated by these observations, we employ a thiol self-assembly strategy using 1-dodecanethiol (DDT) to introduce a molecular-level, thickness-minimized hydrophobic layer anchored through chemisorbed Ag–S bonding. This ionomer-free approach enables precise control over interfacial wettability while minimizing transport penalties, allowing clearer isolation of the intrinsic Ag–Cu tandem contribution. Furthermore, the degree of surface modification can be systematically tuned (e.g., by varying thiol concentration and alkyl-chain length) to identify optimal conditions that simultaneously preserve efficient gas transport and sustain effective tandem operation.

The resulting DDT-Ag/Cu increases the C_**2+**_ partial current density from 225.1 ± 1.94 to 370.5 ± 8.45 mA cm^–2^ and shifts product distribution, raising the ethanol-to-ethylene ratio from ∼0.5 to ∼1.0. These findings show that tuning surface wettability improves gas transport and clarifies how a model Ag/Cu tandem system governs product selectivity under flow-cell CO_2_ electroreduction conditions.

## 2 Experimental Section

### 2.1 Chemicals

1-Dodecanethiol (98%) was purchased from Thermo Scientific. Absolute ethanol (99.8+%) was purchased from Fisher Scientific. KOH (99.9%) was purchased from Applied Science. All chemicals were used without further purification.

### 2.2 Preparation of Cu and Ag/Cu Gas-Diffusion Electrodes

Metallic Cu with a thickness of 300 nm was sputtered on a Carbon paper (AvCarb GDS3250) using a pure Cu target (99.99%) with an Ar flow rate of 3 mTorr at a DC power of 30 W in a magnetron sputtering system for 43 min. The base pressure is below 5 × 10^–6^ Torr, acquired using a turbomolecular pump.

A 100 nm-thick Metallic Ag layer was deposited on the prepared Cu gas-diffusion electrode by electron-beam (e-beam) evaporation in a high-vacuum system. Prior to deposition, the chamber base pressure was maintained at approximately 3 × 10^–6^ Torr. The deposition rate was monitored in situ using a quartz crystal microbalance (QCM) and stabilized at 0.1 Å/s through real-time adjustment of the e-beam power. A relatively low deposition rate was employed to ensure uniform film growth and to suppress excessive surface roughness commonly associated with Ag evaporation. All depositions were carried out at room temperature, and the film thickness was determined by integrating the QCM signal during deposition. The deposition process was conducted at the Taiwan Semiconductor Research Institute (TSRI), Taiwan.

### 2.3 Preparation of the DDT-Ag/Cu Gas-Diffusion Electrode

To prepare the hydrophobically modified electrode, a 1 mM *DDT* solution was prepared by dissolving 4.8 μL of DDT in 20 mL of absolute ethanol. Ten mL of this solution was transferred to a Petri dish, and the Ag/Cu gas-diffusion electrode was placed face-down on the liquid surface for 10 min to allow self-assembly. The electrode was then removed, rinsed thoroughly with ethanol, dried on a hot plate, and finally vacuum-dried to obtain the DDT-Ag/Cu gas-diffusion electrode.

### 2.4 Characterization

Morphology was examined by field-emission scanning electron microscopy (FESEM; JSM-6700F, JEOL). Crystal structure was analyzed by X-ray diffraction (XRD; D8 Advance, Bruker AXS) using Cu Kα radiation (λ = 1.5406 Å). High-resolution X-ray diffraction (XRD) measurements were performed over a 2θ = 30–50° with a step size of 0.02° and a scan rate of 2 min per degree. Static water contact angles were measured with a contact-angle goniometer (PSC-1000B). Surface analysis of X-ray Photoelectron Spectroscopy (XPS; Thermo Fisher Scientific Theta Probe) using Al Kα radiation (1486.6 eV) at the Taiwan Semiconductor Research Institute (TSRI), Taiwan. X-ray absorption spectroscopy (XAS) at the Cu K-edge—including X-ray absorption near edge spectra (XANES) and extended X-ray absorption fine structure (EXAFS) —was collected in fluorescence mode at beamline BL32A of TPS, NSRRC. Spectra were processed by subtracting the pre-edge baseline and normalizing to the postedge region. EXAFS analysis employed applying k^2^-weighting and Fourier transform of χ­(k) to evaluate the contributions of individual scattering pairs to the radial (R-space) peaks.

### 2.5 Electrochemical Measurements

Electrochemical measurements were performed on a Biologic VSP-3e potentiostat in a flow-cell reactor using a gas diffusion electrode (GDE) as the working electrode (WE), nickel foam as the counter electrode (CE), and a saturated Ag/AgCl electrode as the reference electrode (RE). The WE and CE compartments were separated by an anion exchange membrane (AEM). All tests used 1.0 M KOH aqueous solution as the electrolyte, with CO_2_ supplied to the gas chamber at a flow rate of 50 sccm. Potentials were converted to the reversible hydrogen electrode (RHE) scale according to E_RHE_ = E_Ag/AgCl_ + 0.0591 × pH + E^0^_Ag/AgCl_, where E^0^_Ag/AgCl_, where E^0^_Ag/AgCl_ = 0.210 V versus the standard hydrogen electrode (SHE) at 25 °C, and all values were corrected for *iR* drop. Gaseous products were quantified using gas chromatography (GC, Agilent 8860) equipped with a thermal conductivity detector (TCD) and a flame ionization detector (FID). Separation was carried out on an Agilent J&W HP-PLOT Q PT GC column (30 m × 0.32 mm i.d. × 20.0 μm film thickness). The GC oven temperature was maintained at 200 °C during analysis. Liquid products, excluding ethanol, were analyzed by high-performance liquid chromatography (HPLC, Agilent 1260 Infinity II) equipped with a variable-wavelength detector (VWD, 210 nm) and a refractive index detector (RID), using 5 mM H_2_SO_4_ as the mobile phase. Ethanol was also quantified by gas chromatography–mass spectrometry (GC–MS, Agilent 7890A GC coupled to a 5975C MS detector).

### 2.6 Operando Raman Measurements

Operando Raman spectra were acquired on a Renishaw inVia Raman microscope using a 785 nm diode laser and a water immersion objective. Measurements were performed in a modified flow cell in which the anodic and cathodic chambers were separated by an anion exchanged membrane (Fumasep FAA-3-PK-130). CO_2_ with a flow rate of 50 sccm was supplied to the gas chamber throughout the measurements. An saturated Ag/AgCl electrode and a nickel foam served as the reference and counter electrodes, respectively.

## 3 Results and Discussion

To investigate the intrinsic effect of the *tandem* configuration under flow-cell conditions, metallic Ag/Cu electrodes were fabricated by sequential physical-vapor deposition—magnetron sputtering and electron-beam evaporation. This bilayer design establishes a purely physical Ag–Cu interface, eliminating confounding effects from alloying and ionomer binders. A sputtered Cu-only electrode served as the control.

### 3.1 Cu and Ag/Cu

To verify that Ag and Cu are physically mixed rather than alloyed, we examined crystal structure, oxidation state, and local coordination. X-ray diffraction (XRD) patterns (Figure 1a), after subtracting the carbon-paper background, shows fcc Cu reflections at 2θ = 43.3°, 50.4° and 74.1° indexed to (111), (200), and (220) (JCPDS 04–0836), and Ag reflections at 2θ = 38.1°, 44.3° and 64.4°assigned to (111), (200) and (220) (JCPDS 04–0783).^40^ Then, high-resolution X-ray diffraction (XRD) measurements were performed to examine potential interfacial alloying or strain effects, with particular attention to the Ag(111) and Cu(111) reflections (Supplementary Figure S1). No discernible peak shifts are observed after Ag deposition, indicating negligible lattice strain and the absence of Ag–Cu alloying. Synchrotron X-ray absorption spectroscopy (XAS) provides additional evidence for this conclusion. The Cu K-edge XANES spectra of the Cu and Ag/Cu electrodes closely match that of Cu foil (Figure 1b), suggesting that Cu remains predominantly metallic. The Fourier-transformed k^2^-weighted EXAFS (Figure 1c) is dominated by the Cu–Cu shell at ∼2.2 Å (phase-uncorrected), and no obvious features attributable to Ag–Cu scattering are observed within the sensitivity of our measurements. EXAFS fitting of the Cu and Ag/Cu samples (Supplementary Figure S2 and Table S1), calibrated against a Cu foil reference, yields a Cu–Cu coordination numbers of approximately 9 with average bond distances of ∼2.54 Å. These results indicate a predominantly metallic Cu coordination environment, further supporting the formation of a physically assembled Ag/Cu interface rather than an alloyed structure.

**Figure 1:**
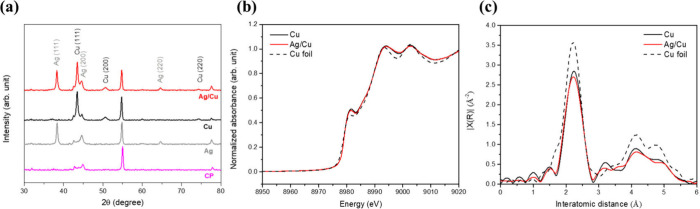
(a) XRD patterns of Cu and Ag/Cu electrodes. (b) Cu K-edge XANES spectra and (c) Fourier-transformed EXAFS spectra

Surface morphology was examined by field-emission scanning electron microscopy (FESEM), as shown in Supplementary Figure S3. The pristine carbon paper (Supplementary Figure S3a) displays the rough, porous texture characteristic of the microporous layer (MPL), which facilitates gas diffusion during CO_2_RR. After Cu sputtering (Supplementary Figure S3b), a dense, uniform metallic coating conformally covers the MPL. Subsequent Ag evaporation yields an Ag/Cu electrode with a similar topography (Supplementary Figure S3c), indicating that the deposited Ag film conforms to the underlying Cu texture rather than altering it. Insets in Supplementary Figure S3 present digital photographs highlighting the macroscopic color evolution from black (carbon paper) to reddish-brown (Cu) and silvery-gray (Ag/Cu) following metal deposition.

In CO_2_RR tests, the Ag/Cu electrode delivered catalytic performance and product distributions that were essentially indistinguishable from those of the Cu-only electrode (Figure 2). The Cu electrode reached its optimum performance at a current density of 300 mA cm^–2^, delivering a C_**2+**_ Faradaic efficiency (FE) of 75.02 ± 0.65%, while the Ag/Cu electrode reached a comparable FE of 76.02 ± 0.18% under the same conditions. Across a wide current-density range, no discernible enhancement in CO selectivity was observed for the Ag/Cu electrode.

**Figure 2:**
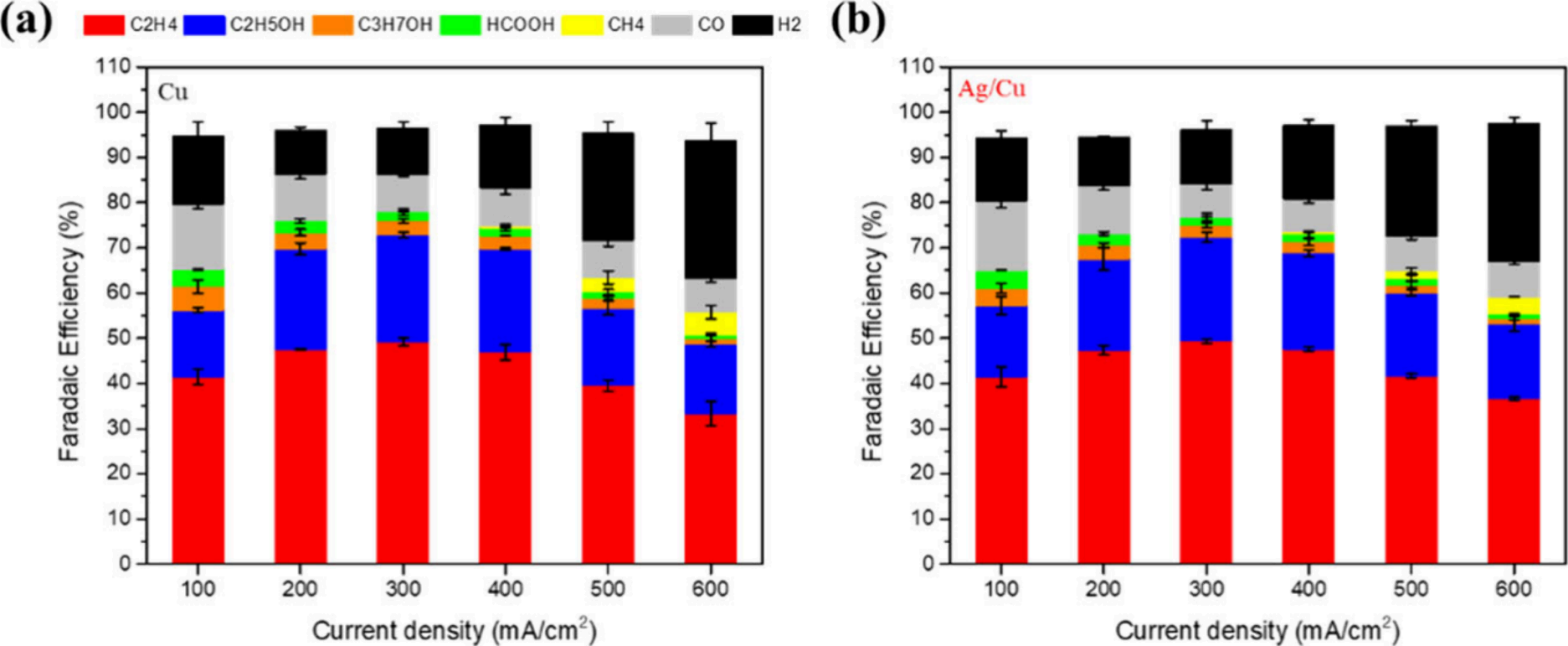
CO_2_RR performance of (a) Cu and (b) Ag/Cu electrodes at different current densities. Error bars represent the mean ± standard deviation (SD) based on three independent measurements (*n* = 3).

Given that electrode thickness can influence catalytic performance in flow-cell CO_2_RR, we performed control experiments in which the Cu thickness was fixed while the Ag thickness was systematically varied (10, 30, 50, 200, and 300 nm). As shown in Supplementary Figure S4, both the Faradaic efficiencies and product distributions exhibit only marginal variations across this range, suggesting that Ag thickness is not the dominant factor governing the observed selectivity trends in this system.

In contrast, reversing the deposition sequence (Ag/Cu vs Cu/Ag) leads to a modest increase in CO selectivity (Supplementary Figure S5). Notably, however, this increase in CO formation does not translate into an enhanced C_**2+**_ fraction in our configuration, implying that CO generated on the Ag layer is not efficiently transferred to or utilized by the overlying Cu. Taken together, these control experiments support that the conclusion that the suppressed CO_2_RR activity of Ag under ionomer-free conditions limits its effective contribution to CO production, which is consistent with the absence of a measurable tandem effect.

### 3.2 DDT-Ag/Cu

To explore the origin of the suppressed tandem effect, we noted that for both Cu and Ag/Cu electrodes, the H_2_ Faradaic efficiency rose sharply once the applied current density exceeded 300 mA cm^–2^. Such an increase in hydrogen evolution reaction (HER) is a hallmark of electrode flooding, implying electrolyte breakthrough at high current densities. To examine wetting behavior, we conducted contact-angle measurements on bare carbon paper (Supplementary Figure S6) and on the Ag/Cu electrode (Figure 3a). Metal deposition markedly increased surface hydrophilicity, even when applied to inherently hydrophobic carbon paper. SEM analysis further reveals that the Ag/Cu electrode surface undergoes pronounced surface restructuring after the reaction (Supplementary Figure S7). This restructuring is likely induced by excessive electrolyte penetration, which can cover or block Ag active sites.^32^ These observations suggest that the hydrophilic overlayer promotes electrolyte infiltration, obstructing CO_2_ transport pathways and thereby suppressing Ag participation in CO production, ultimately undermining the intended Ag–Cu tandem catalysis.

**Figure 3:**
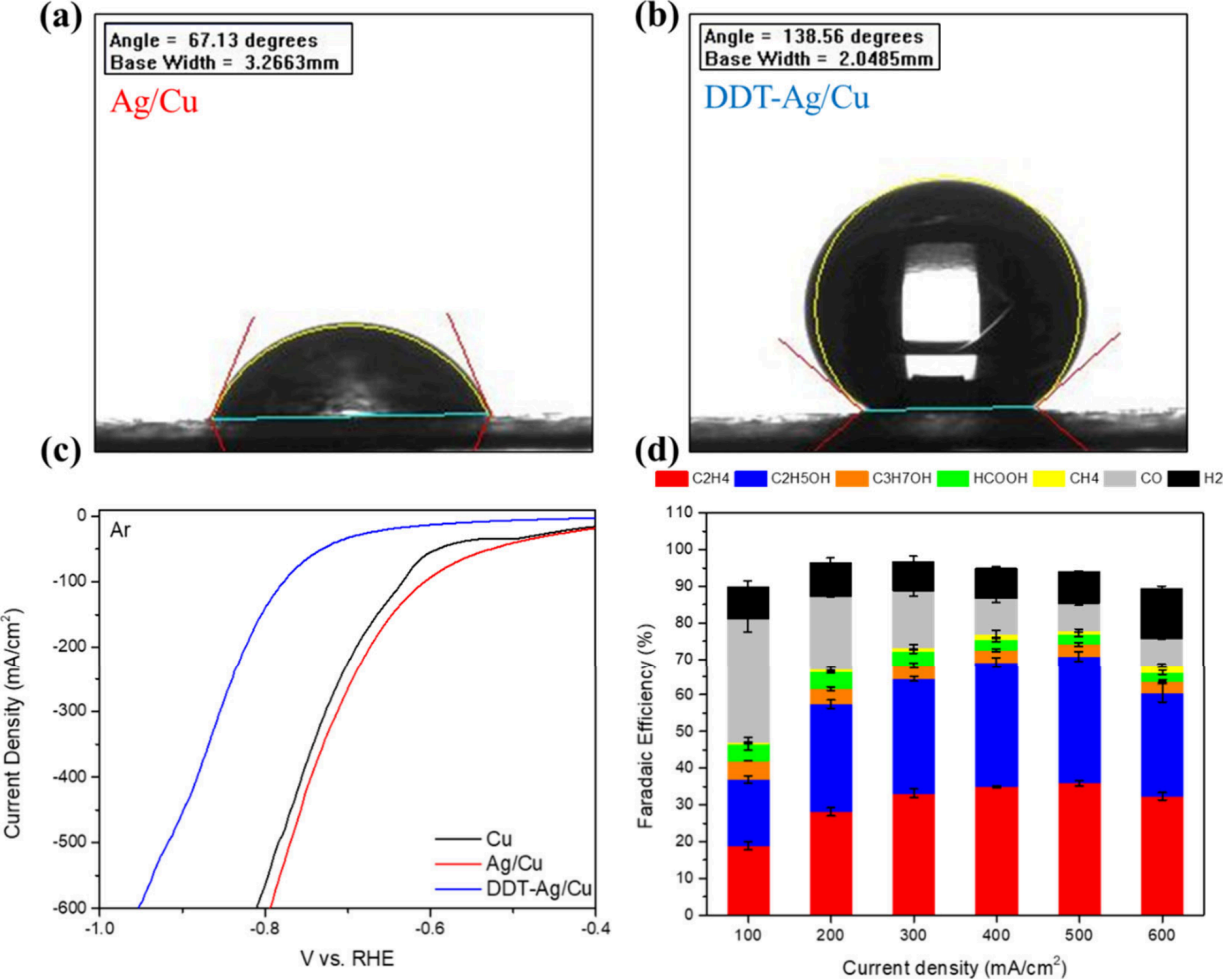
(a) Contact angle of Ag/Cu, (b) after DDT modification, (c) LSV in Ar, and (d) CO_2_RR performance of DDT–Ag/Cu. Error bars represent the mean ± standard deviation (SD) based on three independent measurements (*n* = 3).

To mitigate electrode flooding, a hydrophobic surface modification was introduced using 1-dodecanethiol (DDT). Owing to the strong affinity of thiol groups for Ag surfaces, DDT rapidly self-assembles via chemisorbed Ag–S bonding. In this configuration, the thiol headgroup anchors to Ag, while the saturated alkyl chain extends outward to enhance surface hydrophobicity, yielding the DDT–Ag/Cu electrode. After modification, the static contact angle increased from 67.13° to 138.56° (Figure 3b), confirming substantially enhanced hydrophobicity. To assess whether flooding was alleviated, linear sweep voltammetry (LSV) was conducted in Ar environment to emulate HER-prone conditions (Figure 3c). Whereas Cu and Ag/Cu exhibit higher HER activity, DDT–Ag/Cu shows a larger HER overpotential and markedly suppressed hydrogen evolution, demonstrating the effectiveness of the hydrophobic strategy.

The electrode retained their morphological and structural integrity after modification. FESEM imaging (Supplementary Figure S8a) shows no discernible change in surface morphology, while the digital image inset reveals a brighter, more silvery appearance. XRD patterns (Supplementary Figure S8b) exhibit no peak shifts or new reflections, indicating unchanged phase composition and lattice parameters. Supplementary Figure S8c,d and Figure S8e,f present the cross-sectional SEM images and corresponding EDX elemental mappings of Ag/Cu and DDT–Ag/Cu, respectively. In both samples, the bilayer Ag/Cu architecture is clearly preserved and conformally supported on the porous carbon substrate, indicating that the DDT modification does not disrupt the layered structure. Taken together, these results confirm that the DDT treatment modulates surface wettability without altering either the chemical composition or crystal structure of the electrodes.

To evaluate scalability and modification uniformity, DDT treatment was further applied to a larger Ag/Cu-GDE (9 × 9 cm^2^) using the same immersion protocol (10 min), as shown in Supplementary Figure S9. Although ethanol is volatile, the short immersion time allows the process to be carried out in a relatively sealed container, thereby minimizing solvent loss and concentration drift. Importantly, XPS measurements collected from multiple locations (center and four corners) of the 9 × 9 cm^2^ electrode consistently exhibit detectable S signals, indicating spatially uniform modification across the substrate. These results demonstrate that the DDT self-assembly process is experimentally practical and exhibits strong potential for scalable electrode preparation.

CO_2_RR performance was then evaluated (Figure 3d and Supplementary Figure S10–S11). As shown in (Supplementary Figure 10a,b), FE­(CO) and FE­(H_2_). The DDT–Ag/Cu electrode maintained a low H_2_ Faradaic efficiency (<10%) across 100–600 mA cm^–2^. Concurrently, the CO Faradaic efficiency increased significantly at lower current densities. As a reference, a 100 nm thermally evaporated Ag electrode exhibits a water contact angle comparable to that of bare carbon paper (Supplementary Figure S11a), which is likely due to the limited thickness of the Ag film and the dominant influence of the porous substrate. Across the tested current-density range, this Ag-only electrode maintains a nearly constant CO Faradaic efficiency of ∼80% (Supplementary Figure S11b,c), confirming its robust intrinsic activity for CO_2_-to-CO conversion.

In contrast, the DDT–Ag/Cu electrode shows a higher CO partial current density at low current densities, followed by a gradual decrease as the total current density increases (Supplementary Figure S11d). Notably, this decrease coincides with enhanced multicarbon formation on Cu, suggesting that CO generated on the Ag layer is increasingly and efficiently consumed by the underlying Cu layer via the tandem pathway to produce C_**2+**_ products at high current densities. At a totally current density of 500 mA cm^–2^, DDT–Ag/Cu achieved a maximum C_2+_ FE of 74.09 ± 1.69% with a C_2+_ partial current density (*J*_C2+_) of 370.5 ± 8.45 mA cm^–2^. We further examined the morphology of the DDT–Ag/Cu electrode after reaction. As shown in Supplementary Figure S12a, the postreaction SEM image remains highly comparable to that of the pristine electrode (Supplementary Figure S12b) This structural integrity suggests that the Ag/Cu bilayer interface is largely preserved under the tested conditions, which is consistent with the restored tandem behavior observed for DDT–Ag/Cu. We attribute this restoration to the enhanced interfacial hydrophobicity, which improves gas transport and mitigates electrolyte intrusion, thereby maintaining effective CO generation/transport on Ag and subsequent utilization on Cu during flow-cell CO_2_RR.

Postreaction XRD analysis of Ag/Cu and DDT–Ag/Cu (Supplementary Figure S12c) shows no obvious changes in the diffraction patterns compared with the pristine electrodes, with the Ag- and Cu-related reflections remaining at essentially the same positions and intensities. This result indicates that, regardless of possible surface reconstruction at the nanoscale, the electrodes retain a heterophase Ag/Cu architecture rather than forming a detectable bulk alloy phase.

XPS further confirmed the successful thiol modification (Supplementary Figure S13). In the S 2p region, the modified electrode exhibits significant sulfur components at ∼162 eV and ∼163 eV, attributed to surface-bound thiolate species and Ag–S bonds, respectively. After operating at a constant current of 500 mA cm^–2^, the S 2p signal remains detectable, indicating that the DDT layer is at least partially retained on the electrode surface and is not completely removed under the applied reaction conditions.

### 3.3 Comparison of C2 Products

To further evaluate the *tandem* effect beyond the increased CO Faradaic efficiency at low current densities, we analyzed product selectivity. As shown in Figure 4a, the ethanol-to-ethylene ratio (FE_C_2_H_5_OH_/FE_C_2_H_4__) remains ∼0.5 for both Cu and Ag/Cu across current densities, whereas DDT–Ag/Cu raises this ratio to ∼1.0. Corresponding to LSV under CO_2_ atmosphere (Supplementary Figure S14), we constructed potential-dependent FE plots for ethylene (FE_C_2_H_4__–V, Figure 4b) and ethanol (FE_C_2_H_5_OH_–V, Figure 4c) are visualized. Over a broad potential window, DDT–Ag/Cu exhibits consistently lower FE_C_2_H_4__ but higher FE_C_2_H_5_OH_ than Cu, indicating that the Ag/Cu *tandem* interface intrinsically favors ethanol formation. The hydrophobic DDT modification primarily restores—and amplifies—this *tandem* behavior by improving CO_2_/CO transport and sustaining Ag-mediated CO delivery to the underlying Cu layer.

**Figure 4:**
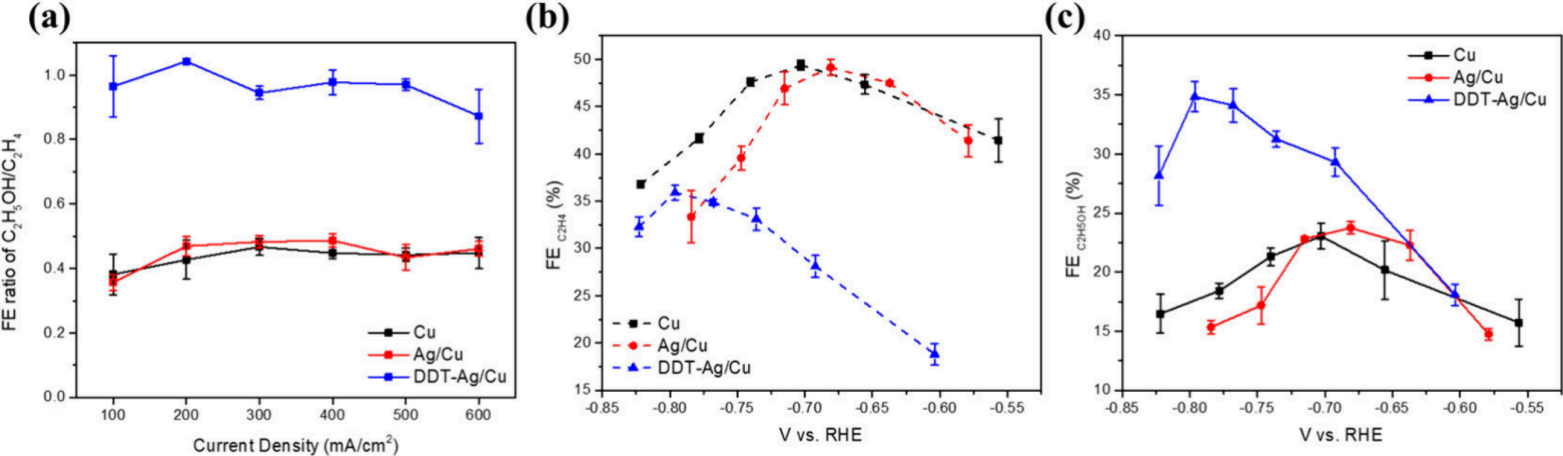
(a) ethanol-to-ethylene ratio at different current densities. (b) FE_C_2_H_4__–V curve, and (c) FE_C_2_H_5_OH_–V curve.

To evaluate whether DDT modification directly alters product selectivity on Cu, we prepared a DDT-functionalized Cu electrode (DDT–Cu) using the same modification protocol. As shown in Supplementary Figure S15a, DDT–Cu exhibits an increased water contact angle, confirming enhanced surface hydrophobicity. Despite this change, the overall product distribution remains ethylene-dominant (Supplementary Figure S15b), and the FE­(CO) at low current densities does not increase (Supplementary Figure S15c). Notably, DDT–Cu still shows suppressed HER (Supplementary Figure S15d), while the ethanol-to-ethylene ratio remains approximately constant at ∼0.5. (Supplementary Figure S15e) Collectively, these results indicate that DDT modification has a limited impact on the intrinsic Cu selectivity under our conditions. Accordingly, the restored selectivity shift observed for DDT–Ag/Cu is therefore more consistent with an interfacial or tandem effect rather than a direct ligand effect on Cu.

### 3.4 Optimization of Modification Conditions

We further compared thiols with different alkyl-chain lengths—1-hexanethiol (MCN) and 1-octadecanethiol (ODT)—as control modifiers. As shown in Supplementary Figure S16a, MCN leads to relatively higher CO production at low current densities; however, its HER suppression is less effective than that of DDT, yielding a C_**2+**_ partial current density of 297.88 mA cm^–2^. By In contrast, the ODT-modified electrode shows a noticeable increase in methane formation (Supplementary Figure S16b) accompanied by a reduced C_**2+**_ partial current density of 212.7 mA cm^–2^. These results indicate that excessively long alkyl chains can unfavorably alter the interfacial microenvironment and mass transport, thereby diminishing tandem performance.

We further investigated the influence of DDT concentration on catalytic performance by modifying electrodes with DDT solutions at 0, 0.2, 0.6, 1.0, 1.4, 1.8, 5.0, and 10 mM, followed by product analysis at 500 mA cm^–2^. As summarized in Supplementary Figure S17, excessively high DDT concentrations lead to diminished CO_2_RR performance accompanied by increased methane formation. This trend is consistent with the possibility that excessive surface coverage perturbing the interfacial microenvironment and mass-transport properties. In contrast, low DDT concentrations provide insufficient HER suppression, leading to a less favorable selectivity profile. Collectively, these results suggest the existence of an optimal modification regime at intermediate DDT concentrations.

### 3.5 Electrochemical Analysis

Electrochemical impedance spectroscopy (EIS) was employed to assess charge-transfer characteristics during CO_2_RR. Nyquist plots for Cu, Ag/Cu, and DDT–Ag/Cu are shown in Supplementary Figure S18. With increasing applied potential, the semicircle diameter progressively decreases, indicative of catalyst activation and reduced charge-transfer resistance. Notably, the contraction is less pronounced for DDT–Ag/Cu, consistent with the hydrophobic DDT modifiers limiting electrolyte ingress and partially suppressing double-layer charging.

To gain deeper mechanistic insight, we analyzed Bode plots (Supplementary Figure S19), plotting phase angle versus logarithm of frequency. With increasing applied potential, the phase angle decreases, showing the activation of catalytic surface. Noticeable differences in characteristic frequency and magnitude emerge among the three electrodes. Frequency-potential phase contours (Figure 5) reveal that DDT–Ag/Cu exhibited a higher initial characteristic frequency at relatively mild potentials than Cu or Ag/Cu, indicating faster initial interfacial reactions—likely associated with CO generation—and aligning with the higher CO FE at low current densities. At more negative potentials, the Cu and Ag/Cu responses nearly vanish beyond −0.55 V vs RHE, whereas DDT–Ag/Cu maintains a strong and broadened response over a wider potential window. These results indicate that the hydrophobic modification enhances gas transport and sustains coupled charge and mass transfer at high current densities and thereby reactivating tandem catalysis.

**Figure 5:**
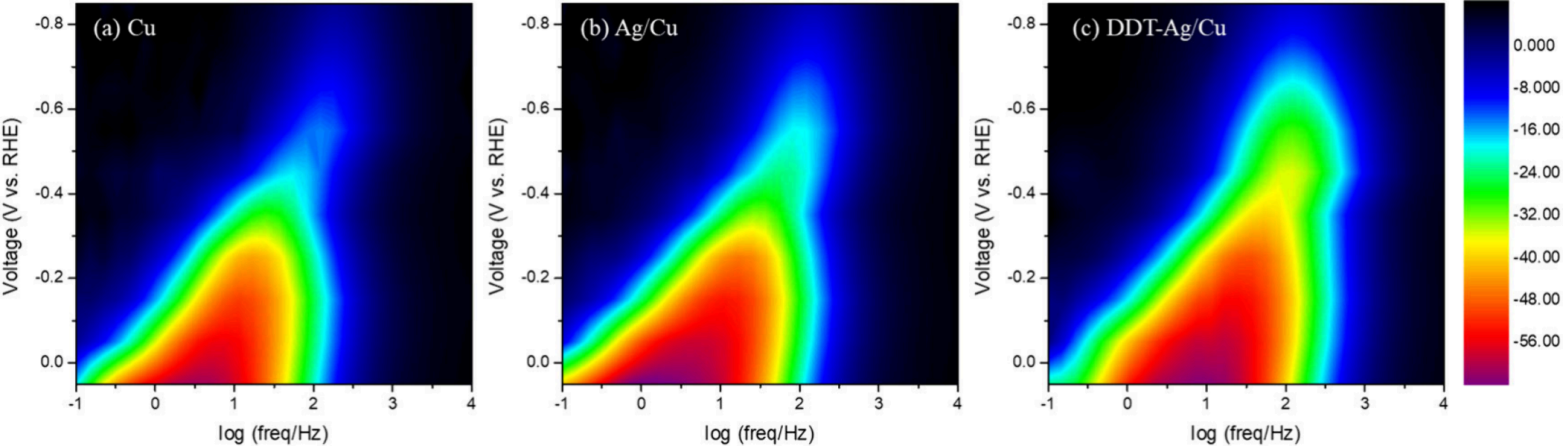
Two-dimensional Bode phase maps of (a) Cu, (b) Ag/Cu, and (c) DDT–Ag/Cu electrodes under different potentials.

To further investigate interfacial redox characteristics, cyclic voltammetry (CV) was performed on Ag/Cu and DDT–Ag/Cu, as shown in Figure 6. In the CV profiles, the redox peaks in the range of 1.0–1.7 V vs RHE are assigned to Ag redox processes, whereas those in the range of 0–0.8 V are attributed to Cu redox processes. Compared with Ag/Cu, all of these redox features for DDT–Ag/Cu shift to more negative potentials. These cathodic shifts are attributed to charge redistribution at the metal–self-assembled monolayer (SAM) interface upon alkanethiol adsorption. Saturated alkanethiols are known to establish an interfacial dipole oriented from the metal toward the organic layer, thereby lowering the metal work function.^41^ This dipole arises from electron transfer from surface Ag atoms to thiolate sulfur, resulting in an overall negative shift in the electrochemical potential; thus, the observed cathodic shift in the CV provides supporting electrochemical evidence for successful DDT SAM modification on the Ag surface.

**Figure 6:**
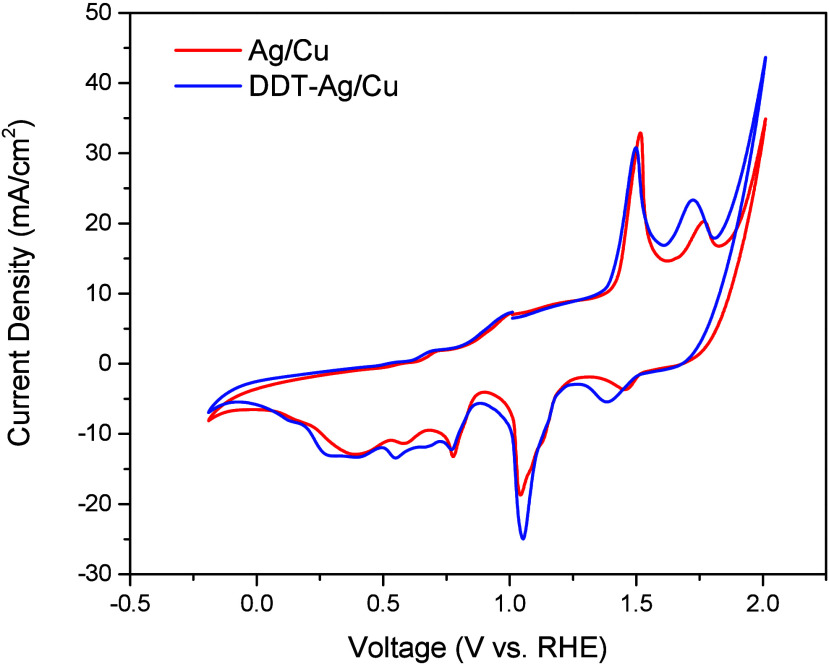
Cyclic voltammograms of Ag/Cu and DDT–Ag/Cu electrodes.

Subsequently, the electrochemical surface area (ECSA) was estimated from the non-Faradaic region of the CV curves (Supplementary Figure S20a,b). The double-layer capacitance (C_dl_)—taken as the slope of capacitive current versus scan rate (Supplementary Figure S20c)—was used as the ECSA proxy. The DDT–Ag/Cu electrode exhibits a markedly smaller C_dl_ (1.69 mF cm^–2^) than Ag/Cu (6.12 mF cm^–2^), indicating that the hydrophobic DDT layer partially blocks electrolyte infiltration and limits formation of the electrochemical double layer.^42–44^ This result is consistent with the Nyquist analysis.

### 3.6 In Situ Raman Spectroscopic Analysis

To probe key intermediates during CO_2_RR, in situ Raman spectroscopy was performed on Ag/Cu and DDT–Ag/Cu electrodes, as shown in Figure 7. At open-circuit potential (OCV), both electrodes exhibited characteristic peaks at ∼530 cm^–1^ and ∼620 cm^–1^, which can be attributed to surface CuO_*x*_ species; these signals rapidly vanished once a potential was applied.^45^ Spectra were Subsequently acquired at five applied potentials from −0.4 V to −0.8 V vs RHE.

**Figure 7:**
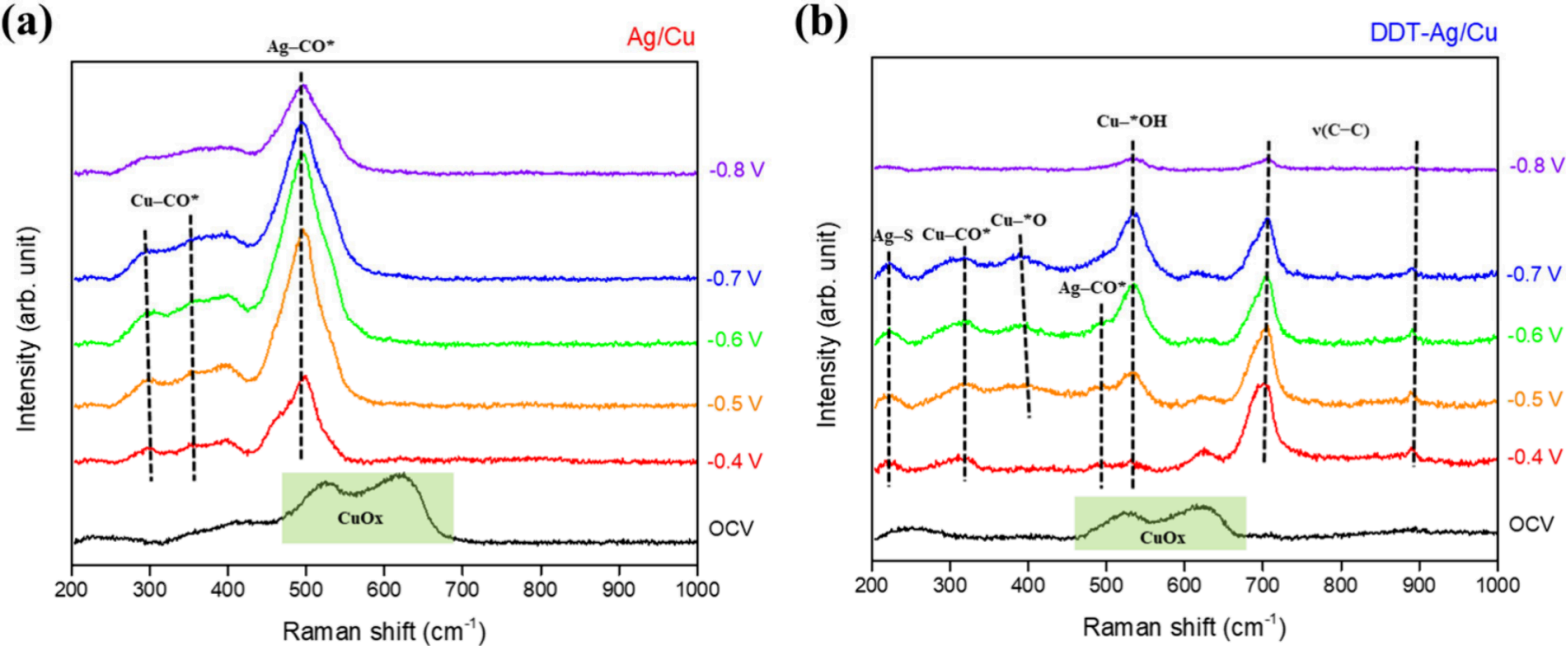
In situ Raman spectra of (a) Ag/Cu and (b) DDT–Ag/Cu electrodes, spectral region from 200 to 1000 cm^–1^.

Compared with Ag/Cu, the DDT–Ag/Cu electrode exhibits three additional, persistent Raman peaks at ∼220, 700, and 890 cm^–1^. The ∼220 cm^–1^ feature is assigned to Ag–S stretch, while the 700 and 890 cm^–1^ peaks correspond to ν­(C–C) stretching mode of the surface DDT layer.^46,47^ These signals diminish sharply at potentials more negative than −0.8 V vs RHE, likely due to thiolate desorption, in line with the observed CO_2_RR performance trends.

Beyond the ∼300 and 360 cm^–1^ peaks observed on both electrodes—assigned to Cu–CO* stretching and frustrated rotating,^48–50^ —the Ag/Cu sample exhibits a strong and persistent Raman feature in the 450–540 cm^–1^ range, attributed to Ag–CO* adsorption.^36,51^ This feature remains prominent even at −0.8 V, suggesting that CO* formed on Ag accumulates and is not efficiently transferred to, or consumed by, downstream reactions on Cu. In contrast, for DDT–Ag/Cu, the Ag–CO* feature reaches a maximum intensity near −0.5 V and subsequently diminishes toward baseline at more negative potentials. This behavior is consistent with increasingly rapid CO* consumption during C–C coupling on the underlying Cu as the tandem pathway becomes operative. Meanwhile, the 400 and 530 cm^–1^ peaks—assigned to Cu-*O and Cu–*OH vibrations—intensify,^45,50,52^ implying enhanced OH^–^ adsorption. We attribute this to the hydrophobic surface, increasing local alkalinity and thereby promoting C–C coupling and the formation of multicarbon products.

We further analyzed the Raman spectra in the 1800–2200 cm^–1^ region (Supplementary Figure S21). No discernible *CO_bridge_ feature near ∼1900 cm^–1^ is detected on DDT–Ag/Cu, whereas both electrodes display the *CO_atop_ band. The *CO_atop_ signal on Ag/Cu is centered at ∼2045 cm^–1^ (LFB-dominant), while DDT–Ag/Cu exhibits a higher-frequency *CO_atop_ band at ∼2089 cm^–1^ (HFB-dominant). Such a shift toward an HFB-dominant *CO_atop_ signature has been reported as a spectroscopic marker correlated with enhanced C_**2+**_ selectivity in Cu-based CO_2_RR systems.^53^

### 3.7 Stability

Finally, we benchmarked the durability of DDT–Ag/Cu against bare Cu under identical flow-cell conditions (Figure 8). Chronopotentiometric tests were conducted at 300 mA cm^–2^ in 1 M KOH, while the Faradaic efficiency (FE) of ethylene was monitored by GC. Compared with Cu, DDT–Ag/Cu exhibits a markedly more stable cell voltage over time, suggesting a more robust interfacial architecture and more sustained gas transport during operation.

**Figure 8:**
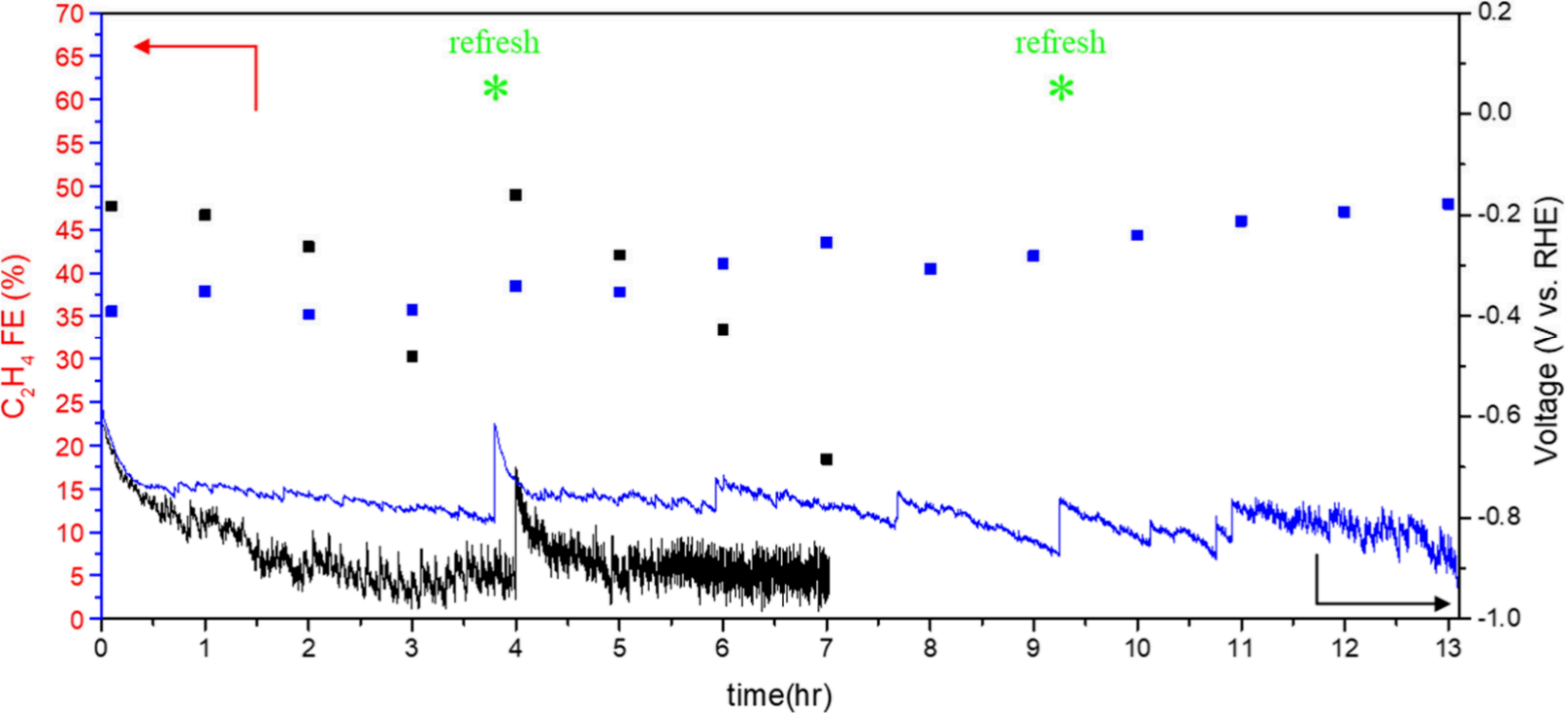
Stability test of DDT-Ag/Cu (blue) and Cu (black).

Although the overall performance of DDT–Ag/Cu remains largely stable throughout the test, occasional electrolyte intrusion into the gas chamber was observed, indicative of partial flooding. In such cases, rinsing the electrode followed by reapplication of the DDT modification restores the initial performance, enabling stable operation for a total cumulative duration of up to 13 h. In contrast, the Cu electrode shows a more rapid decay in FE over time; and postrinsing treatment fails to recover its original stability, pointing to a more irreversible performance degradation under the same conditions.

Notably, the ethylene FE of DDT–Ag/Cu gradually increases during the stability test. Postreaction XPS (Supplementary Figure S22) reveals an increased surface Cu signal, which suggests potential interfacial restructuring where Cu progressively covers (or becomes more exposed on) the Ag layer. Such a compositional evolution would shift selectivity toward ethylene, consistent with trends reported in prior MEA studies on Ag–Cu interfacial reconstruction.^32^

## 4 Conclusions

In conclusion, by constructing binder-free, physically assembled Ag/Cu bilayers via sequential sputtering/evaporation and verifying the absence of Ag–Cu alloying (XRD/XAS), we isolated the intrinsic role of the tandem interface under flow-cell conditions, showing in Scheme 1. As-deposited Ag/Cu, however, behaved essentially like Cu because the hydrophilic metal overlayer promoted electrolyte breakthrough at ≥300 mA cm^–2^, suppressing Ag-derived CO production and negating tandem catalysis.

**Scheme 1:**
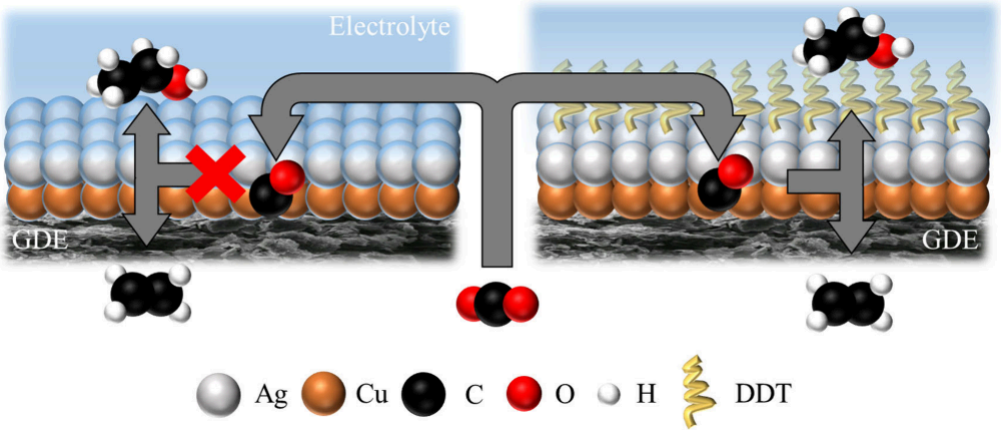
Schematic Comparison of CO_2_ Reduction Pathways on Ag/Cu and DDT–Ag/Cu Electrodes in a Flow Cell System, Highlighting the Role of Hydrophobic Surface Modification in Maintaining Gas Channels for Efficient Tandem Catalysis

Hydrophobizing the Ag surface with a 1-dodecanethiol self-assembled monolayer (DDT–Ag/Cu) reversed this limitation without altering phase or morphology: the static contact angle increased from 67.13° to 138.56°, HER was suppressed, and H_2_ FE remained <10% from 100–600 mA cm^–2^. Under these gas-management conditions, tandem behavior was restored and amplified: at 500 mA cm^–2^ the electrode delivered a C_2+_ FE of 74.09 ± 1.69% with a partial current density of 370.5 ± 8.45 mA cm^–2^, alongside a product redistribution toward ethanol (FE_C_2_H_5_OH_/FE_C_2_H_4__ ≈ 1.0 vs ≈ 0.5 for Cu/Ag-free controls). In situ Raman further shows that, unlike unmodified Ag/Cu where Ag–CO* persists even at −0.8 V, DDT–Ag/Cu exhibits Ag–CO* that peaks near −0.5 V and then vanishes as CO* is consumed in C–C coupling on Cu; concurrent growth of Cu–O/OH bands indicates a locally more alkaline, hydrophobic interface that promotes multicarbon formation.

Collectively, these results identify wettability and gas–liquid management. Surface-energy engineering with simple alkanethiol SAMs provides a general and scalable route to unlock ethanol-rich C_2+_ production at high current density on Ag/Cu and, by extension, other tandem architectures.

## Supplementary Material



## Data Availability

The data supporting this study are available in the paper and the Supporting Information. All other relevant source data are available from the corresponding authors upon reasonable request.

## Notes

Additional XRD patterns; SEM and optical images; XPS and Raman spectra; contact-angle images; linear sweep voltammetry (LSV) data; Faradaic efficiency results; electrochemical impedance spectroscopy (EIS) data (Nyquist and Bode plots); and EXAFS fitting results (PDF)

## References

[1] Overa S., Ko B. H., Zhao Y., Jiao F. (2022). Electrochemical Approaches
for CO_2_ Conversion to Chemicals: A Journey toward Practical
Applications. Acc. Chem. Res..

[2] Nitopi S., Bertheussen E., Scott S. B., Liu X., Engstfeld A. K., Horch S., Seger B., Stephens I. E. L., Chan K., Hahn C., No̷rskov J. K., Jaramillo T. F., Chorkendorff I. (2019). Progress and Perspectives of Electrochemical CO_2_ Reduction on Copper in Aqueous Electrolyte. Chem. Rev..

[3] Segets D., Andronescu C., Apfel U.-P. (2023). Accelerating CO_2_ electrochemical
conversion towards industrial implementation. Nat. Commun..

[4] Xu Z., Lu R., Lin Z.-Y., Wu W., Tsai H.-J., Lu Q., Li Y. C., Hung S.-F., Song C., Yu J. C., Wang Z., Wang Y. (2024). Electroreduction
of CO_2_ to methane with triazole molecular catalysts. Nat. Energy.

[5] Wu Q., Ji S., Chen J., Tan X.-Q., Ong W.-J., Du R., Wang P., Wang H., Qiu Y., Yan K., Zhao Y., Zhao W.-W., Peng K.-S., Chen Y.-Y., Hung S.-F., Zhou L., Wang X., Qiu G., Chen G. (2025). Realizing the practical application of CO_2_ electroreduction
for urban wastewater denitrification. Nat. Water.

[6] Zhang Q., Tsai H. J., Li F., Wei Z., He Q., Ding J., Liu Y., Lin Z.-Y., Yang X., Chen Z., Hu F., Yang X., Tang Q., Yang H. B., Hung S.-F., Zhai Y. (2023). Boosting the
Proton-coupled
Electron Transfer via Fe–P Atomic Pair for Enhanced Electrochemical
CO_2_ Reduction. Angew. Chem., Int.
Ed..

[7] Nguyen T. N., Dinh C. T. (2020). Gas diffusion electrode
design for electrochemical
carbon dioxide reduction. Chem. Soc. Rev..

[8] Malkhandi S., Yeo B. S. (2019). Electrochemical
conversion of carbon dioxide to high
value chemicals using gas-diffusion electrodes. Curr. Opin. Chem. Eng..

[9] Hung S.-F. (2020). Electrochemical
flow systems enable renewable energy industrial chain of CO_2_ reduction. Pure Appl. Chem..

[10] Weekes D. M., Salvatore D. A., Reyes A., Huang A., Berlinguette C. P. (2018). Electrolytic
CO_2_ Reduction in a Flow Cell. Acc.
Chem. Res..

[11] Li L., Wen J., Lo T. W. B., Yin J., Lei Q. (2025). Wettability-Controlled
Electrocatalytic Carbon Dioxide Reduction. Chem.
Methods.

[12] Lin Y., Wang T., Zhang L., Zhang G., Li L., Chang Q., Pang Z., Gao H., Huang K., Zhang P., Zhao Z.-J., Pei C., Gong J. (2023). Tunable CO_2_ electroreduction to ethanol and ethylene with
controllable
interfacial wettability. Nat. Commun..

[13] Hori, Y. Electrochemical CO2 Reduction on Metal Electrodes. Springer New York: pp 89–189.

[14] Wang X., Hu Q., Li G., Yang H., He C. (2022). Recent Advances and
Perspectives of Electrochemical CO_2_ Reduction Toward C_2+_ Products on Cu-Based Catalysts. Electrochem.
Energy Rev..

[15] Wu F.-Y., Tsai H.-J., Lee T.-J., Lin Z.-Y., Peng K.-S., Chen P.-H., Hiraoka N., Liao Y.-F., Hu C.-W., Hsu S.-H., Lu Y.-R., Hung S.-F. (2023). Copper–barium-decorated
carbon-nanotube composite for electrocatalytic CO_2_ reduction
to C_2_ products. J. Mater. Chem. A.

[16] Xu Y., Li C., Xiao Y., Wu C., Li Y., Li Y., Han J., Liu Q., He J. (2022). Tuning the Selectivity of Liquid
Products of CO_2_RR by Cu–Ag Alloying. ACS Appl. Mater. Interfaces.

[17] Xu A., Hung S.-F., Cao A., Wang Z., Karmodak N., Huang J. E., Yan Y., Sedighian Rasouli A., Ozden A., Wu F.-Y., Lin Z.-Y., Tsai H.-J., Lee T.-J., Li F., Luo M., Wang Y., Wang X., Abed J., Wang Z., Nam D.-H., Li Y. C., Ip A. H., Sinton D., Dong C., Sargent E. H. (2022). Copper/alkaline earth metal oxide
interfaces for electrochemical
CO_2_-to-alcohol conversion by selective hydrogenation. Nat. Catal..

[18] Jin C., Lin Y., Wang Y., Shi J., Li R., Liu Y., Yue Z., Leng K., Zhao Y., Wang Y., Han X., Qu Y., Bai J. (2025). Engineering Atom-Scale Cascade Catalysis
via Multi-Active
Site Collaboration for Ampere-Level CO_2_ Electroreduction
to C_2+_ Products. Adv. Mater..

[19] Du R., Li T., Wu Q., Wang P., Yang X., Fan Y., Qiu Y., Yan K., Wang P., Zhao Y., Zhao W.-W., Chen G. (2022). In Situ Engineering
of the Cu+/Cu0 Interface to Boost C_2+_ Selectivity in CO_2_ Electroreduction. ACS Appl. Mater.
Interfaces.

[20] Shen Y.-J., Hsu Y.-H., Chang Y.-C., Ma J.-J., Peng K.-S., Lu Y.-R., Hsu S.-H., Hung S.-F. (2025). Microenvironment
Matters: Copper–Carbon Composites Enable a Highly Efficient
Carbon Dioxide Reduction Reaction to C_2_ Products. ACS Appl. Mater. Interfaces.

[21] Wang H., Wu Q., Du R., Chen G. (2025). Tuning *CO Adsorption via Cu^+^/Cu^0^ Interface
Engineering for Enhanced Ethylene
Selectivity in Electrochemical CO2 Reduction. ACS Appl. Mater. Interfaces.

[22] Wu Q., Du R., Wang P., Waterhouse G. I. N., Li J., Qiu Y., Yan K., Zhao Y., Zhao W.-W., Tsai H.-J., Chen M.-C., Hung S.-F., Wang X., Chen G. (2023). Nanograin-Boundary-Abundant
Cu_2_O-Cu Nanocubes with High C_2+_ Selectivity
and Good Stability during Electrochemical CO_2_ Reduction
at a Current Density of 500 mA/cm^2^. ACS Nano.

[23] Ma J., Liu C., Bai M., Fu Z., Zhao P., Gao Y., Zhao M., He Y., Xiao H., Jia J. (2023). Recent advances
in application of tandem catalyst for electrocatalytic CO_2_ reduction. Mol. Catal..

[24] Zhang B., Wang L., Li D., Li Z., Bu R., Lu Y. (2022). Tandem strategy for electrochemical CO_2_ reduction
reaction. Chem. Catal..

[25] Lu Y.-H., Shen Y.-J., Tsai H.-J., Lee Y.-H., Huang Y.-Y., Lin Z.-Y., Huang W.-Y., Lee T.-J., Chen G.-L., Hiraoka N., Ishii H., Liu H.-J., Hsu S.-H., Chang C.-C., Xu A., Hung S.-F. (2026). Model thiophene-decorated
nickel porphyrins for tandem CO_2_ reduction. Nat. Synth..

[26] Chen C., Li Y., Yu S., Louisia S., Jin J., Li M., Ross M. B., Yang P. (2020). Cu-Ag Tandem Catalysts
for High-Rate
CO_2_ Electrolysis toward Multicarbons. Joule.

[27] Sun Q., Zhao Y., Tan X., Jia C., Su Z., Meyer Q., Ahmed M. I., Zhao C. (2023). Atomically Dispersed
Cu–Au Alloy for Efficient Electrocatalytic Reduction of Carbon
Monoxide to Acetate. ACS Catal..

[28] Zhang J., Pham T. H. M., Ko Y., Li M., Yang S., Koolen C. D., Zhong L., Luo W., Züttel A. (2022). Tandem effect
of Ag@C@Cu catalysts enhances ethanol selectivity for electrochemical
CO_2_ reduction in flow reactors. Cell
Rep. Phys. Sci..

[29] Luan P., Dong X., Liu L., Xiao J., Zhang P., Zhang J., Chi H., Wang Q., Ding C., Li R., Li C. (2024). Selective Electrosynthesis of Ethanol via Asymmetric
C–C Coupling in Tandem CO_2_ Reduction. ACS Catal..

[30] Wang M., Fang M., Liu Y., Chen C., Zhang Y., Jia S., Wu H., He M., Han B. (2025). Enhanced Intermediates
Inter-migration on Ag Single-Atom Alloys for Boosting Multicarbon
Product Selectivity in CO_2_ Electroreduction. J. Am. Chem. Soc..

[31] Bian L., Bai Y., Chen J.-Y., Guo H.-K., Liu S., Tian H., Tian N., Wang Z.-L. (2025). Hierarchical Tandem Catalysis Promotes
CO Spillover and Trapping for Efficient CO_2_ Reduction to
C_2+_ Products. ACS Nano.

[32] Zhang S., Zhang B., Yang S., Shao T., Li X., Cao R., Cao M. (2025). Nanoscale
Cu–Ag Heterostructures for CO_2_ Reduction to C_2+_ Products. ACS Appl. Nano Mater..

[33] Zhang Z., Fang Q., Yang X., Zuo S., Cheng T., Yamauchi Y., Tang J. (2025). Additives-Modified
Electrodeposition
for Synthesis of Hydrophobic Cu/Cu_2_O with Ag Single Atoms
to Drive CO_2_ Electroreduction. Adv.
Mater..

[34] Tian H., Yang J.-T., Wang X., Jiao H., Gao Z.-F., Zhu K.-Y., He Q., Wang Z.-L. (2025). Ionic liquid-TiO_2_-CuOx composite interfaces
combined with gas directional transmission
for enhanced electrooxidation of methane to ethanol. Appl. Catal. B Environ..

[35] Chen J.-Y., Yang J.-T., Han Y.-S., Huang Y.-Q., Tian N.-N., Li J.-H., Wang Z.-L. (2025). Passivation Engineering toward Integrated
Acidic Oxygen Evolution Electrodes with Stable Catalytic Activity
for over 3000 h. ACS Catal..

[36] Gao X., Jiang Y., Liu J., Shi G., Yang C., Xu Q., Yun Y., Shen Y., Chang M., Zhu C. (2024). Intermediate-regulated
dynamic restructuring at Ag-Cu biphasic interface
enables selective CO_2_ electroreduction to C_2+_ fuels. Nat. Commun..

[37] Ozden A., Li F., García de Arquer F. P., Rosas-Hernández A., Thevenon A., Wang Y., Hung S.-F., Wang X., Chen B., Li J., Wicks J., Luo M., Wang Z., Agapie T., Peters J. C., Sargent E. H., Sinton D. (2020). High-Rate and Efficient
Ethylene Electrosynthesis Using
a Catalyst/Promoter/Transport Layer. ACS Energy
Lett..

[38] Chang M., Ren W., Ni W., Lee S., Hu X. (2023). Ionomers Modify the
Selectivity of Cu-Catalyzed Electrochemical CO_2_ Reduction. ChemSusChem.

[39] Chang Q., Zhang G., Chen J., Du X., Wang C., Cai Y., Du Y., Zhang P., Wang T., Gong J. (2025). Construction
of efficient electrodes for CO_2_RR through microenvironment
regulation of hydrophobic ionomer. J. Energy
Chem..

[40] Wu X., Chen F., Jin Y., Zhang N., Johnston R. L. (2015). Silver–Copper
Nanoalloy Catalyst Layer for Bifunctional Air Electrodes in Alkaline
Media. ACS Appl. Mater. Interfaces.

[41] Rusu P. C., Giovannetti G., Brocks G. (2007). Dipole Formation at Interfaces of
Alkanethiolate Self-assembled Monolayers and Ag(111). J. Phys. Chem. C.

[42] Campiña J. M., Martins A., Silva F. (2009). Probing the Organization of Charged
Self-Assembled Monolayers by Using the Effects of pH, Time, Electrolyte
Anion, and Temperature on the Charge Transfer of Electroactive Probes. J. Phys. Chem. C.

[43] Cheng Y., Li Q., Salaman M. I. B., Wei C., Wang Q., Ma X., Liu B., Wong A. B. (2025). Microenvironment Tailoring for Electrocatalytic
CO_2_ Reduction: Effects of Interfacial Structure on Controlling
Activity and Selectivity. J. Am. Chem. Soc..

[44] Chen G., Ge L., Ma B., Kuang Y., Rabiee H., Dorosti F., Nanjundan A. K., Zhu Z., Wang H. (2025). Pore Accessibility
Matters in CO_2_ Electrolysis: Preventing H_2_ Formation
and Boosting Triple-Phase Boundary on Microtubular Gas-Diffusion Electrodes. Appl. Catal. B: Environ. Energy.

[45] Chang X., Zhao Y., Xu B. (2020). pH Dependence of Cu
Surface Speciation
in the Electrochemical CO Reduction Reaction. ACS Catal..

[46] Leverette C. L., Shubert V. A., Wade T. L., Varazo K., Dluhy R. A. (2002). Development
of A Novel Dual-Layer Thick Ag Substrate for Surface-Enhanced Raman
Scattering (SERS) of Self-Assembled Monolayers. J. Phys. Chem. B.

[47] Sadovnikov S. I., Vovkotrub E. G., Rempel A. A. (2018). Micro-Raman Spectroscopy of Nanostructured
Silver Sulfide. Dokl. Phys. Chem..

[48] Park J., Jeong C., Na M., Oh Y., Lee K.-S., Yang Y., Byon H. R. (2024). Subnanometer Cu
Clusters on Porous
Ag Enhancing Ethanol Production in Electrochemical CO2 Reduction. ACS Catal..

[49] Zhan C., Dattila F., Rettenmaier C., Bergmann A., Kühl S., García-Muelas R., López N., Cuenya B. R. (2021). Revealing the CO
Coverage-Driven C–C Coupling Mechanism for Electrochemical
CO_2_ Reduction on Cu_2_O Nanocubes via Operando
Raman Spectroscopy. ACS Catal..

[50] Moradzaman M., Mul G. (2021). In Situ Raman Study
of Potential-Dependent Surface Adsorbed Carbonate,
CO, OH, and C Species on Cu Electrodes During Electrochemical Reduction
of CO_2_. ChemElectroChem..

[51] Cai Z., Cao N., Zhang F., Lv X., Wang K., He Y., Shi Y., Bin Wu H., Xie P. (2023). Hierarchical Ag-Cu interfaces promote
C-C coupling in tandem CO_2_ electroreduction. Appl. Catal., B.

[52] Niaura G. (2000). Surface-enhanced
Raman spectroscopic observation of two kinds of adsorbed OH–
ions at copper electrode. Electrochim. Acta.

[53] Shen Y., Chen F., Lin H., Zhao B., Wang J., Tan T. (2025). Observation
of metal-organic interphase in Cu-based
electrochemical CO_2_-to-ethanol conversion. Nat. Commun..

