# Supplementary material for: Hydrophobic Surface Modification Enables Tandem Ag/Cu Catalysis for CO2 Electroreduction
Source: ACS Appl Mater Interfaces. 2026 Apr 20;18(17):24306–16. doi: 10.1021/acsami.5c22445 (PMC13154128; doi:10.1021/acsami.5c22445)
Supplement: Supplementary file 1 [file am5c22445_si_001.pdf]

Supporting Information

Hydrophobic Surface Modification Enables Tandem Ag/Cu Catalysis for CO_2 Electroreduction

Yu-Cheng Liu,[1] Kang-Shun Peng,[1] Yu-Jhih Shen,[1] Shao-Hui Hsu,[2] Ching-Hsuan Chou, [1] Ya-Ching Chang,[1] Ming-Hsuan Li,[1] Ying-Rui Lu,[3] Sung-Fu Hung[1,4]*

[1]*Department of Applied Chemistry and Center for Emergent Functional Matter Science, National Yang Ming Chiao Tung University, Hsinchu 300, Taiwan.*

[2]*Taiwan Semiconductor Research Institute, National Applied Research Laboratories, Hsinchu 300, Taiwan.*

[3]*National Synchrotron Radiation Research Center, Hsinchu 300, Taiwan*

[4]*Department of Medicinal and Applied Chemistry, Kaohsiung Medical University, Taiwan.*

Email: sungfuhung@nycu.edu.tw

This file includes:

Supplementary Figures 1 to 22.

Supplementary Tables 1 to 2.

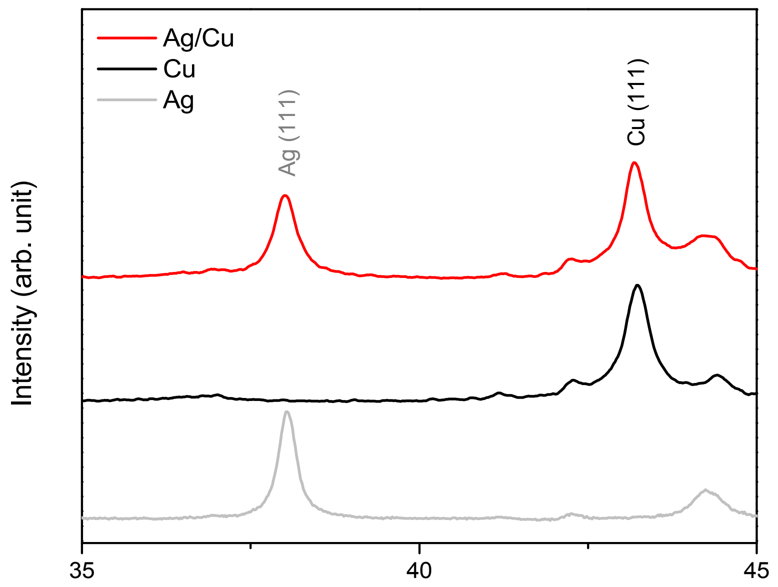

Figure S1. High solution XRD of Ag, Cu, and Ag/Cu

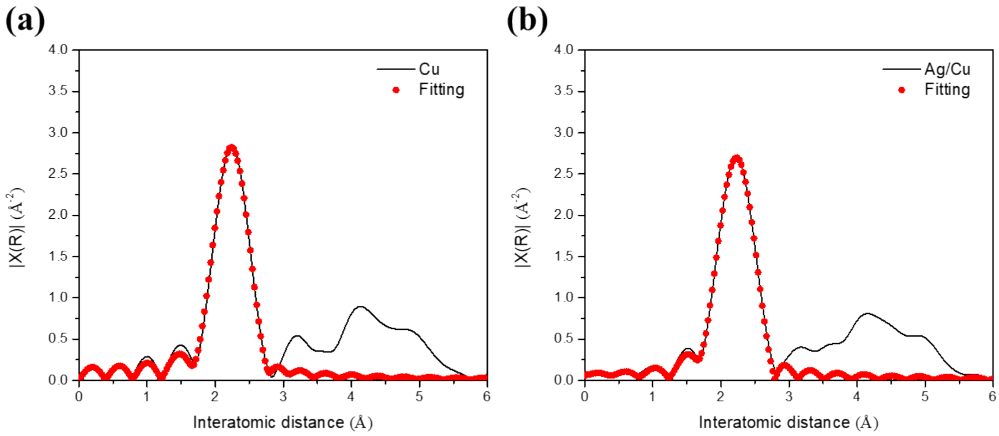

Figure S2. EXAFS fitting of (a) Cu and (b) Ag/Cu.

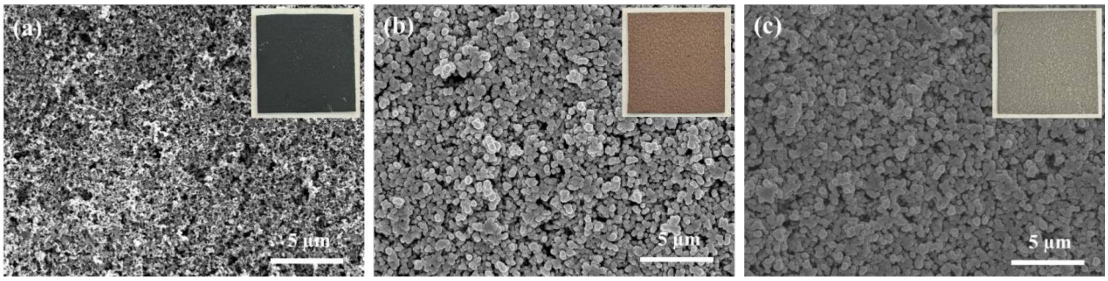

Figure S3. SEM images of (a) carbon paper, (b) Cu, and (c) Ag/Cu electrodes; inset: optical images.

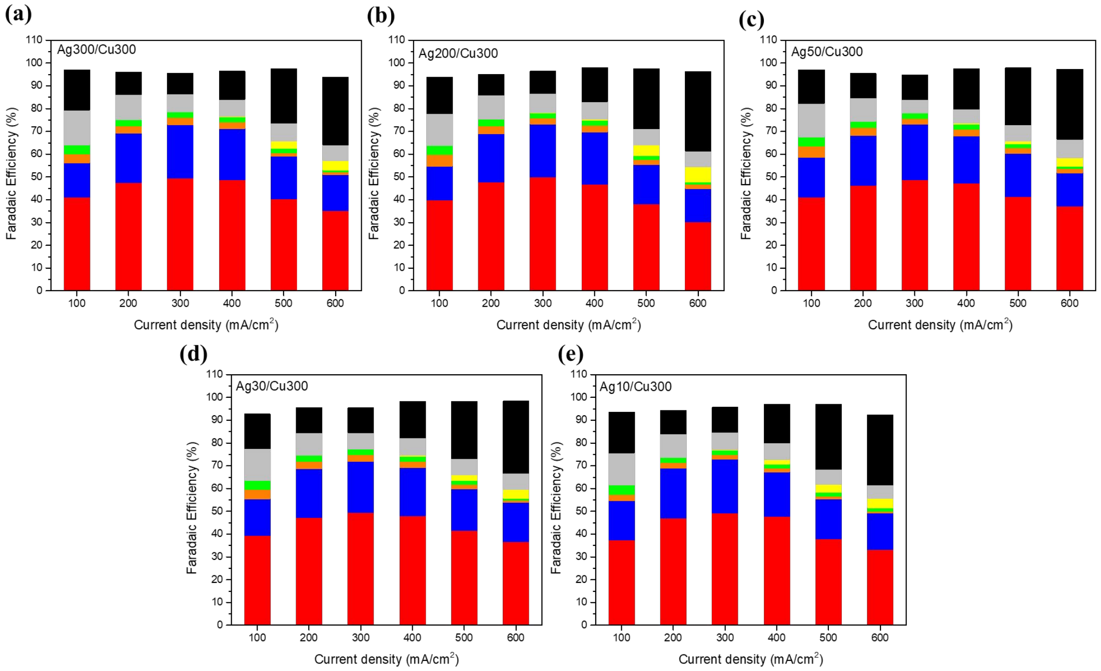

Figure S4. Product distribution of (a) Ag300/Cu300, (b) Ag200/Cu300, (c) Ag50/Cu300, (d) Ag30/Cu300, and (e) Ag10/Cu300.

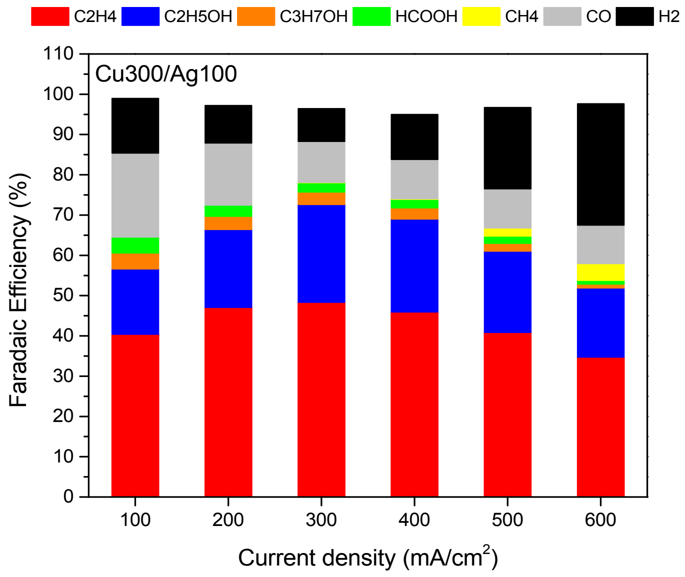

Figure S5. Product distribution of Cu300/Ag100

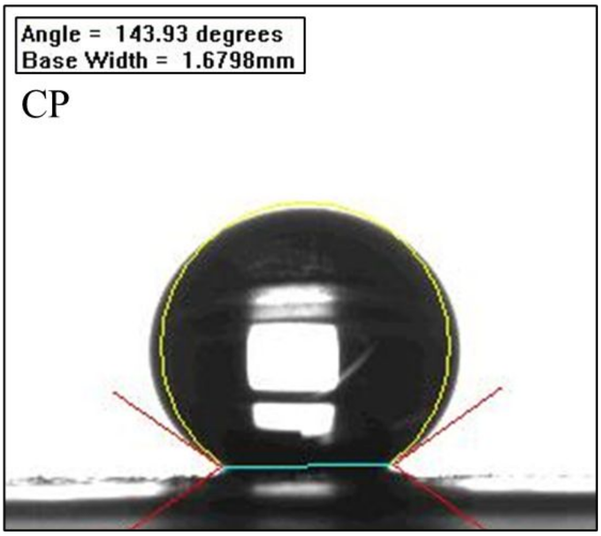

Figure S6. Contact angle of carbon paper.

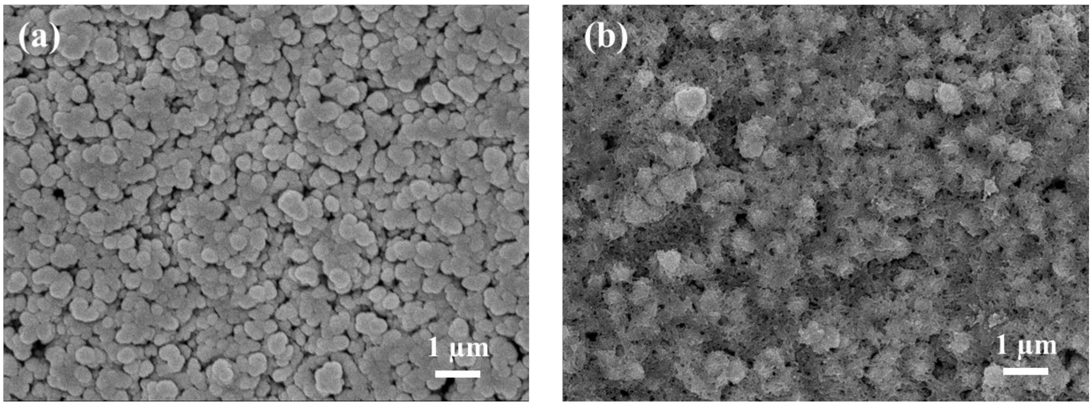

Figure S7. SEM image of Ag/Cu (a) before and (b) after reactions.

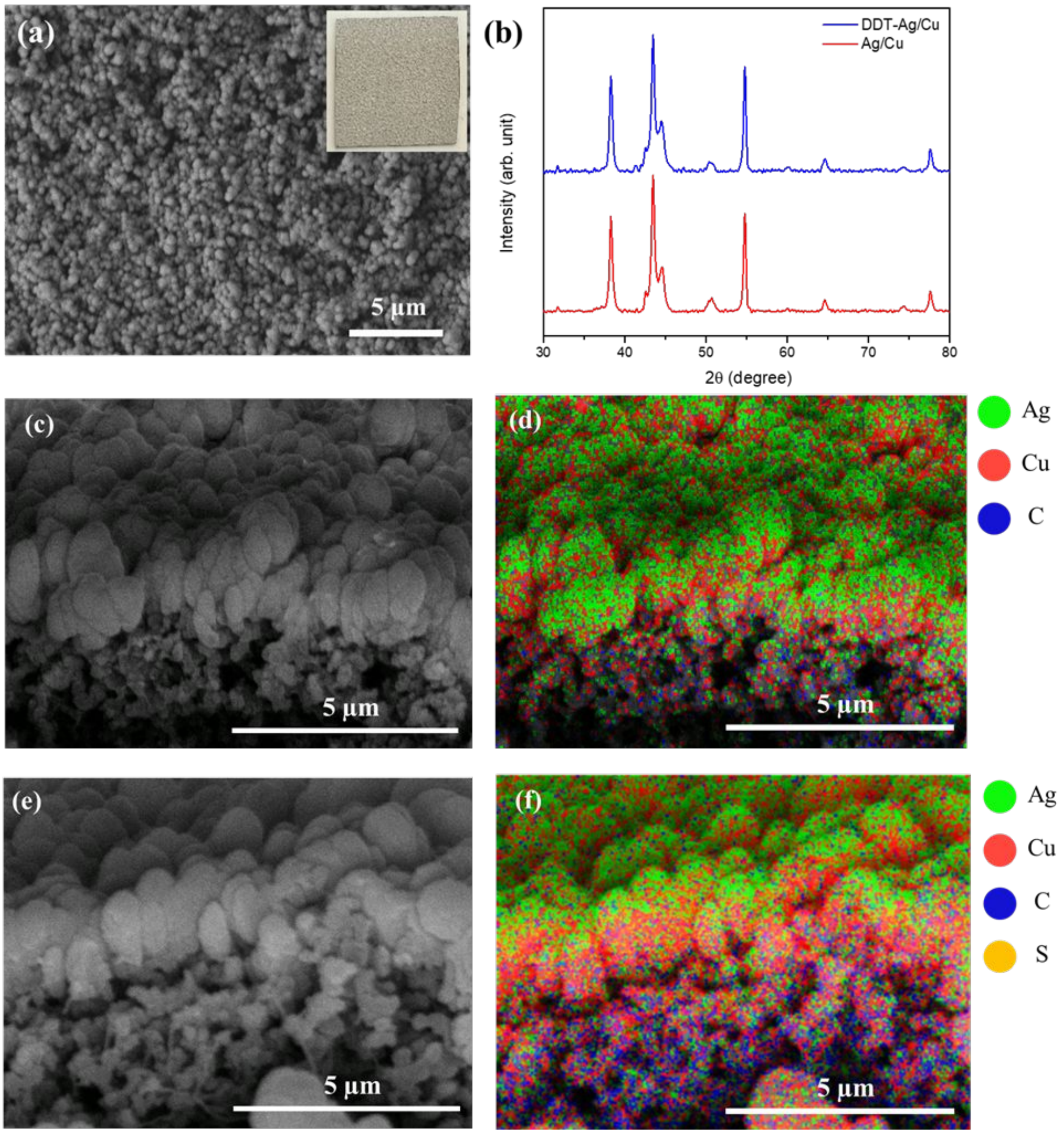

Figure S8. (a) SEM and optical image of DDT-Ag/Cu electrode, (b) XRD of DDT-Ag/Cu electrode, (c) cross-sectional SEM and (d) EDX mapping of Ag/Cu, and (e) cross-sectional SEM and (f) EDX mapping of DDT-Ag/Cu.

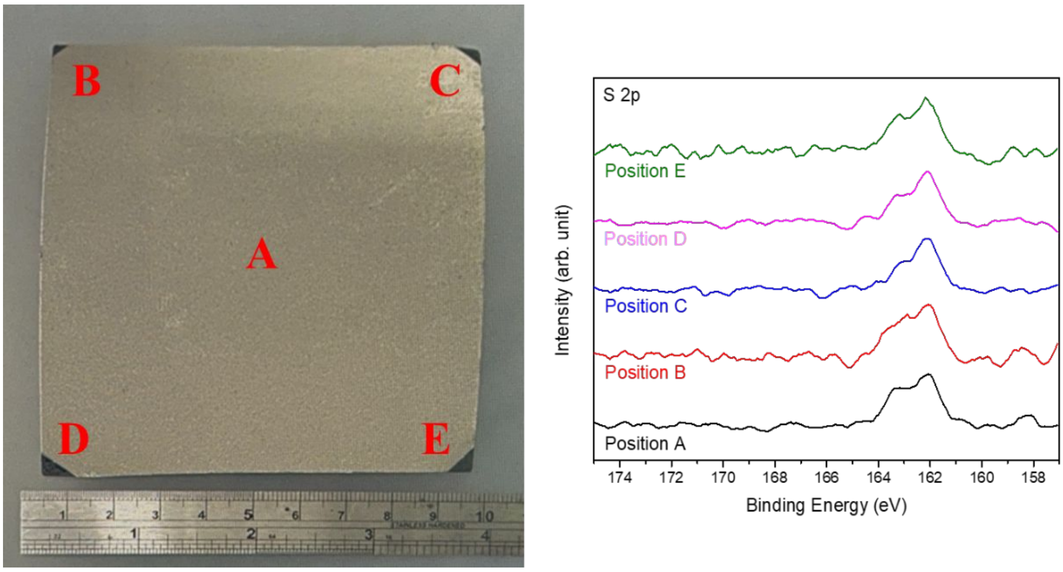

Figure. S9 The evaluation of scalability and modification uniformity. Digital photograph of the large DDT-Ag/Cu-GDE (9 x 9 cm^2) and the XPS measurements collected from multiple locations (center and four corners).

(a) (b)

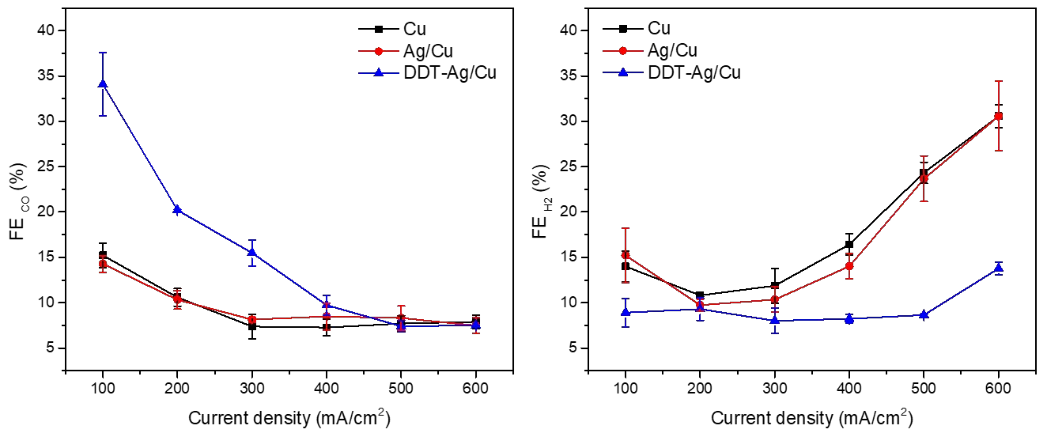

Figure S10. (a) FE(CO) and (b) FE(H_2) of Cu, Ag/Cu, and DDT-Ag/Cu electrodes.

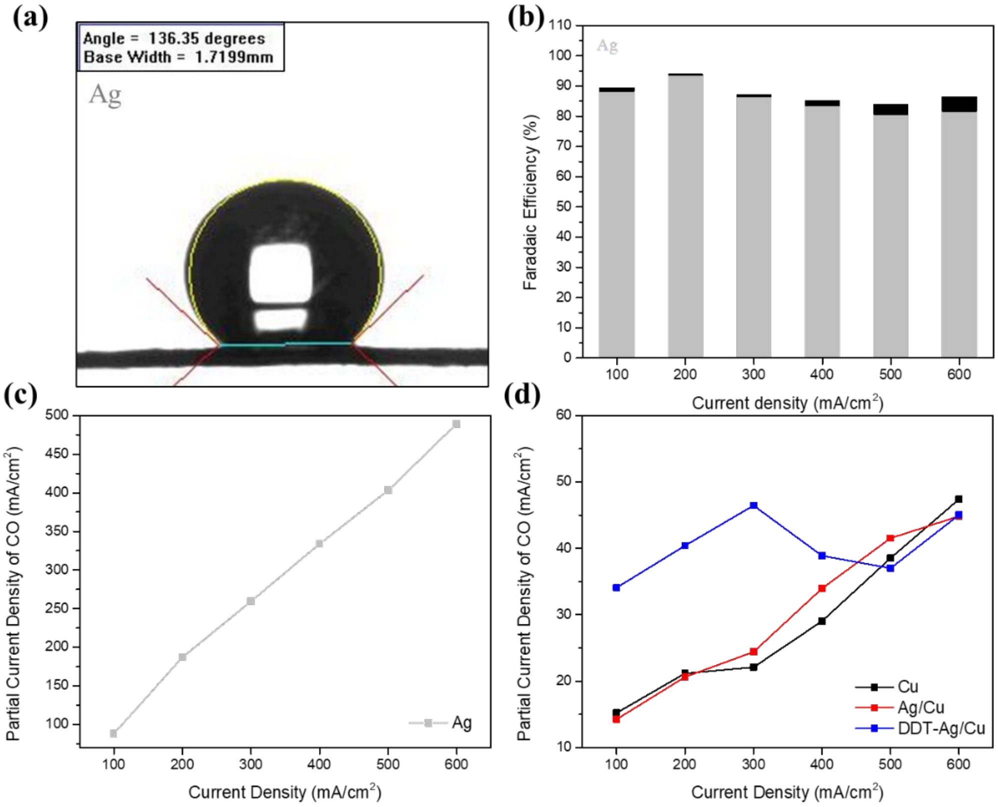

Figure S11. (a) Contact angle of Ag, (b) product distribution of Ag and partial current densities of (c) Ag, (d) Cu, Ag/Cu, and DDT-Ag/Cu.

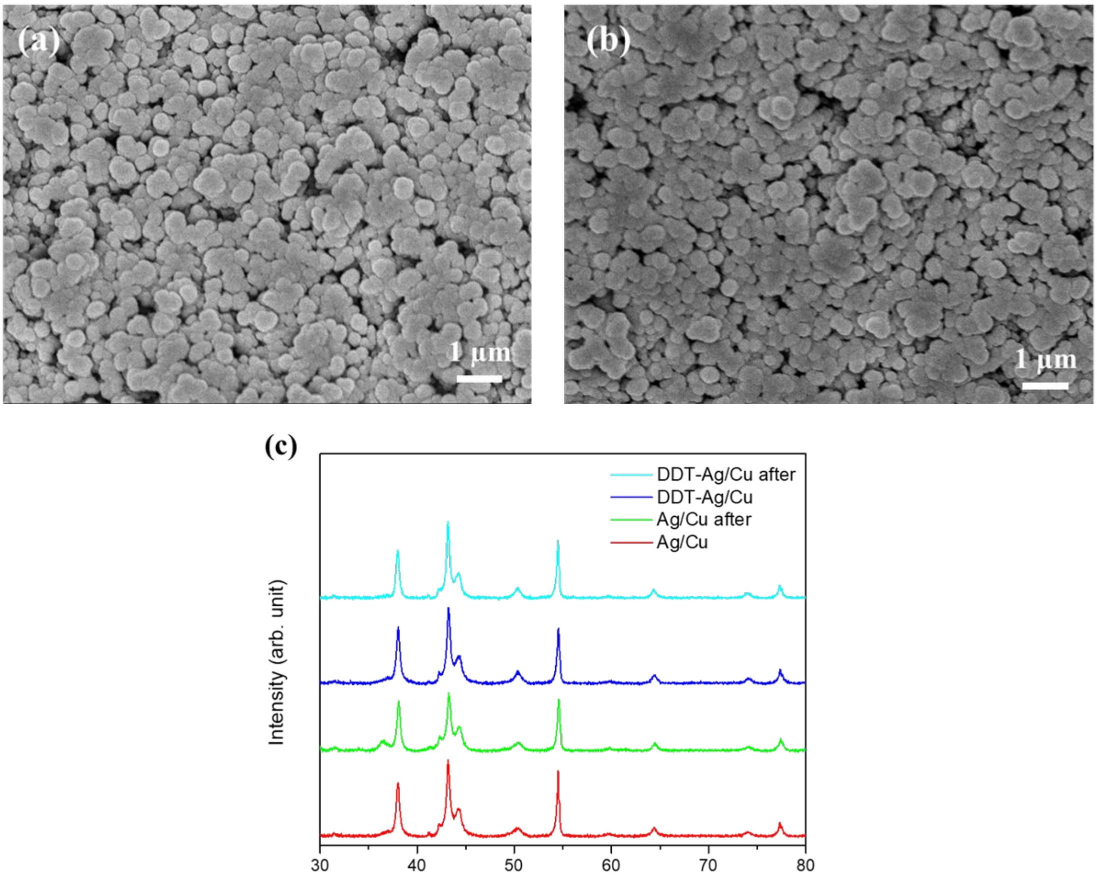

Figure S12. SEM images of DDT-Ag/Cu (a) before and (b) after reactions, and (c) XRD of Ag/Cu and DDT-Ag/Cu before and after reactions.

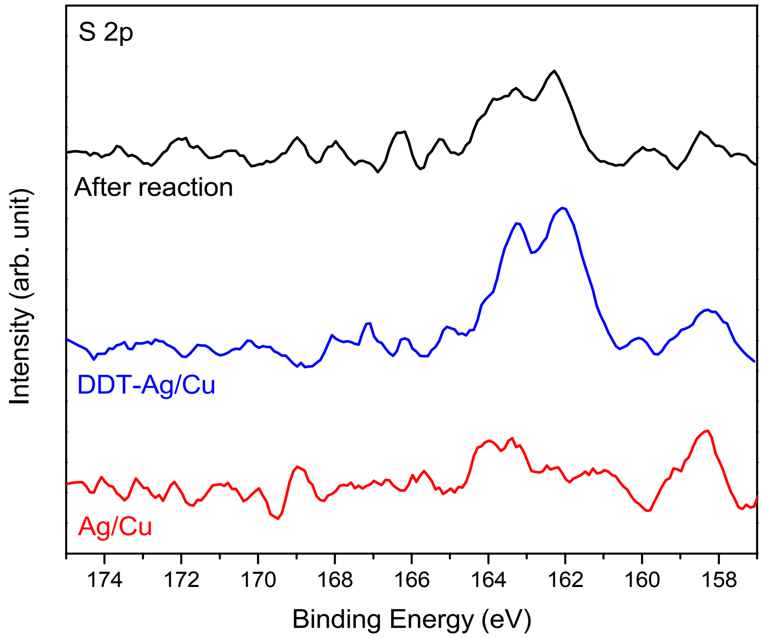

Figure S13. S 2p XPS of Ag/Cu, and DDT-Ag/Cu before and after reactions.

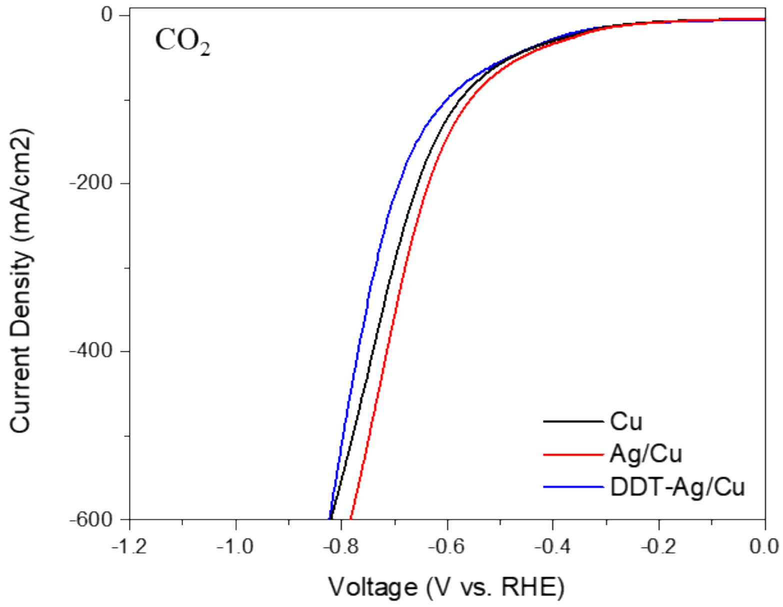

Figure S14. Linear sweep voltammetry (LSV) curves of Cu, Ag/Cu, and DDT–Ag/Cu electrodes under CO_2 condition.

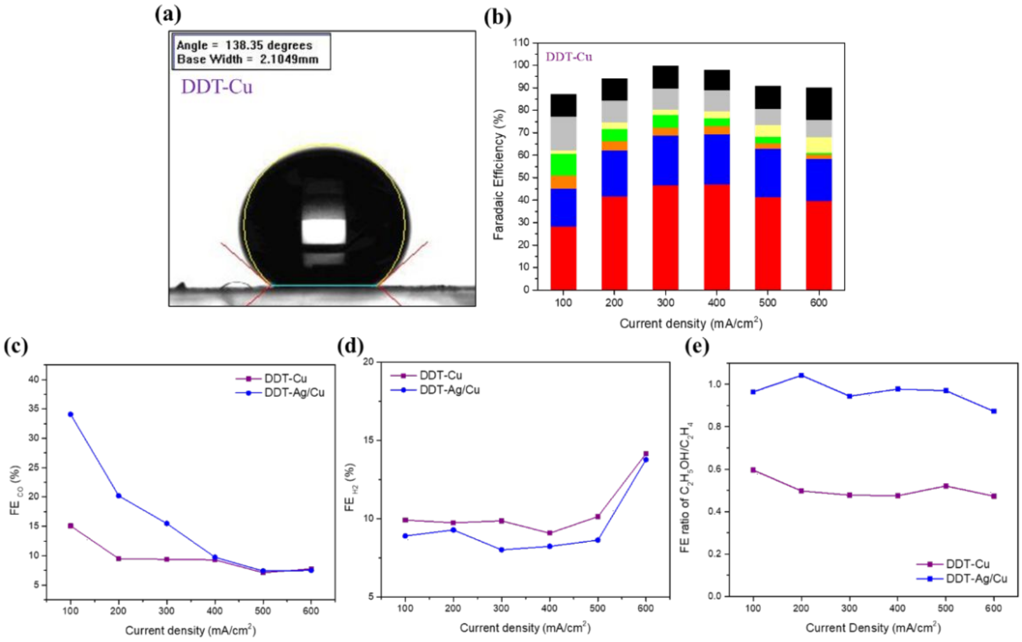

Figure S15. (a) Contact angle of DDT-Cu, (b) Product distribution, (c) FE(CO), (d) FE(H_2), and (e) FE ratio of C_2H_5OH/C_2H_4.

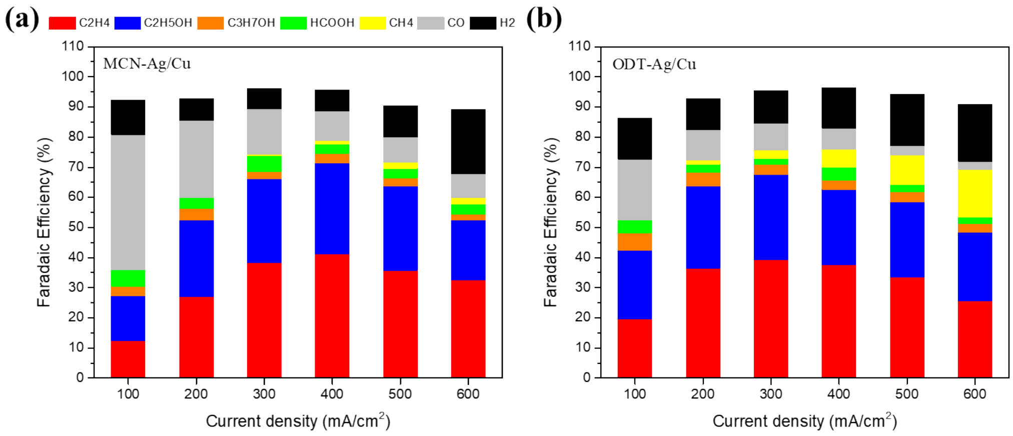

Figure S16. Product distribution of (a) MCN and (b) ODT modification of Ag/Cu electrode.

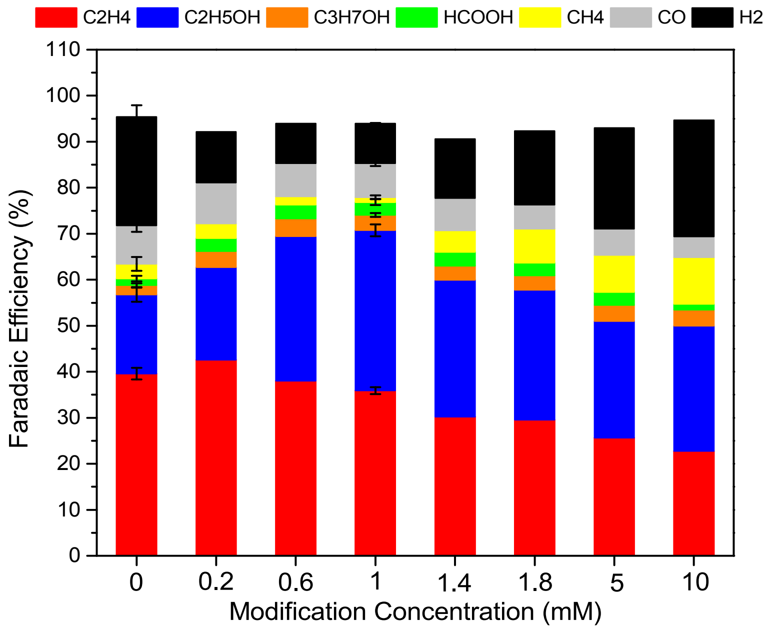

Figure S17. Product distribution of different DDT concentration modified Ag/Cu electrode at 500 mA cm^{-2}.

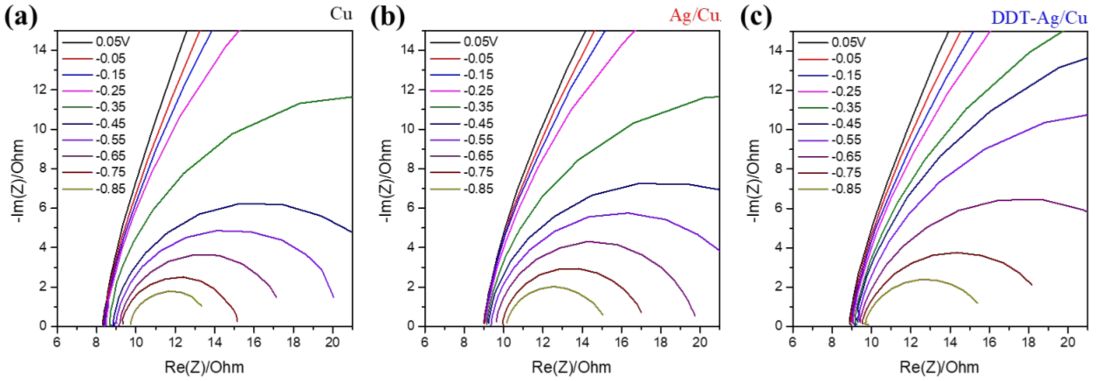

Figure S18. Electrochemical impedance spectroscopy (EIS) Nyquist plots of (a) Cu, (b) Ag/Cu, and (c) DDT–Ag/Cu electrodes at different applied potentials.

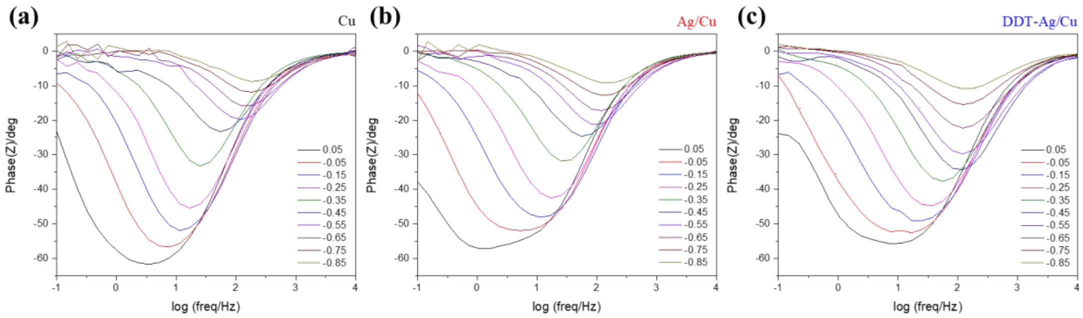

Figure S19. Bode phase plots of (a) Cu, (b) Ag/Cu, and (c) DDT–Ag/Cu electrodes.

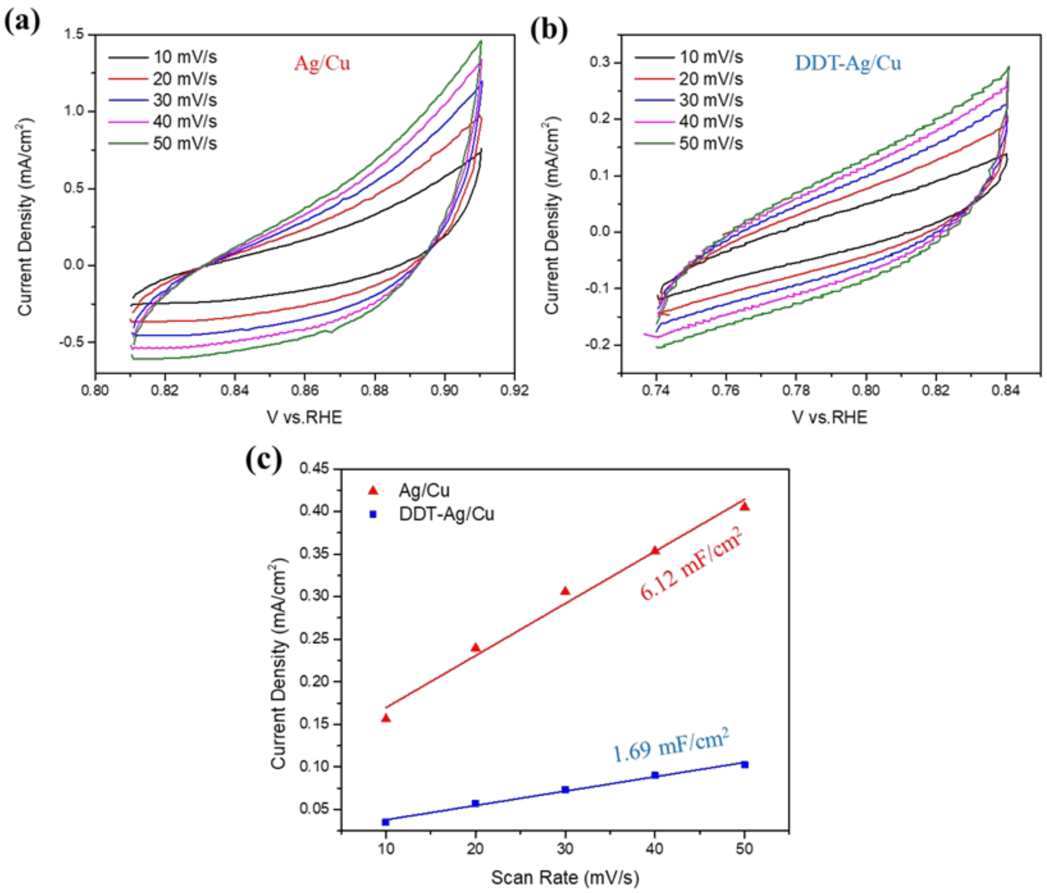

Figure S20. CVs taken over a range of scan rates of (a) Ag/Cu, (b) DDT-Ag/Cu, and (c) corresponding double-layer capacitance plots.

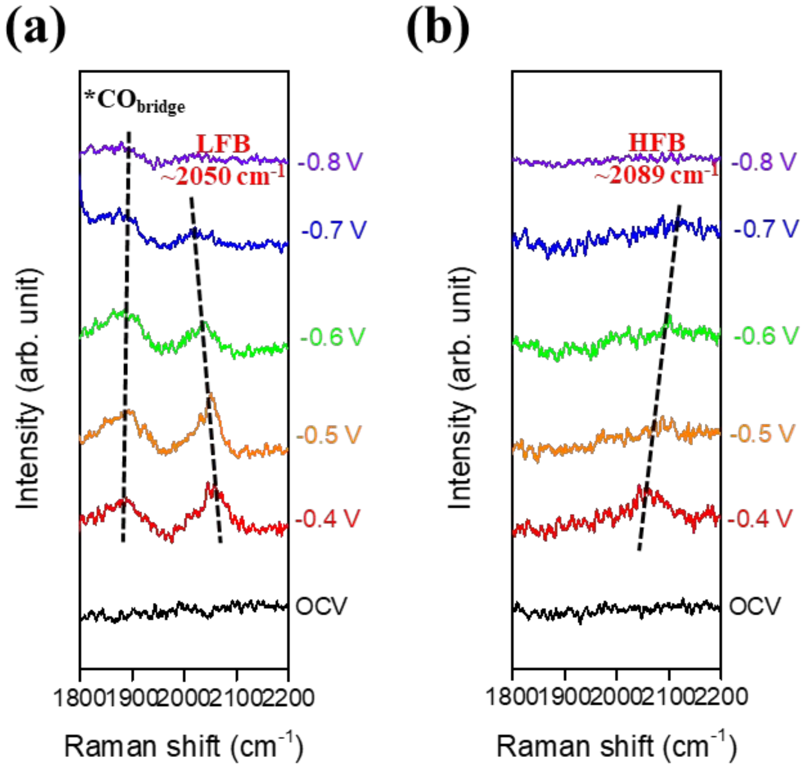

Figure S21. In situ Raman spectra of (a) Ag/Cu and (b) DDT–Ag/Cu electrodes, spectral region from 1900 to 2200 cm^{-1}.

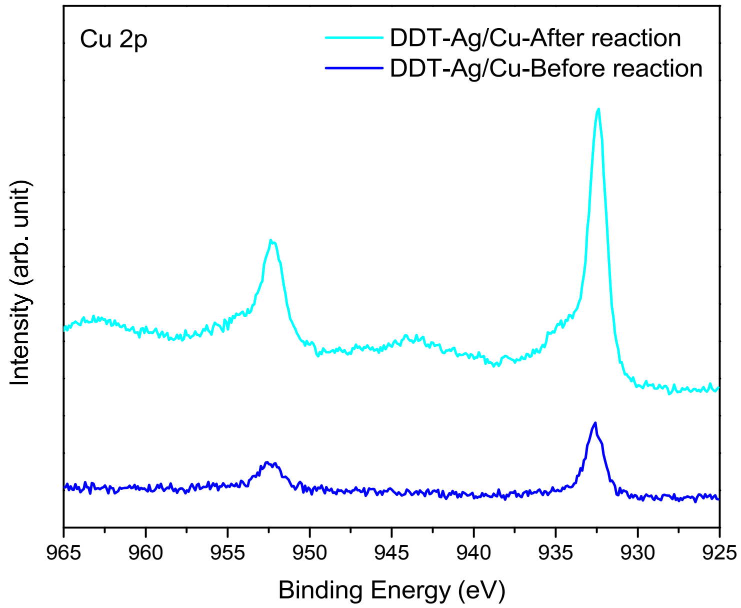

Figure S22. XPS analysis of DDT-Ag/Cu before and after reactions.

Table S1. EXAFS fitting of Cu and Ag/Cu

	Cu	Ag/Cu
Coordination number	9.1 ± 0.9	9.6 ± 1.0
R (Å)	2.54 ± 0.01	2.54 ± 0.01
σ^2	0.007 ± 0.001	0.008 ± 0.001
E_0 (eV)	5.1 ± 0.8	4.7 ± 0.8
R-factor	0.005	0.006

Table S2. Comparison of overall product distribution for CO_2 reduction by Cu、Ag/Cu and DDT-Ag/Cu at various current densities (N.A. = Not Available).

Sample name	Cathode Potential (V vs. RHE)	Current density (mA/cm^2)	C_2H_4 (FE%)	C_2H_5OH (FE%)	C_3H_7OH (FE%)	HCOOH (FE%)	CH_4 (FE%)	CO (FE%)	H_2 (FE%)
Cu	-0.56	100	41.43 ± 2.26	15.73 ± 1.97	3.91 ± 1.06	4.01 ± 0.08	N.A.	15.2 ± 1.36	13.99 ± 1.67
	-0.66	200	47.34 ± 1.02	20.18 ± 2.45	3.08 ± 0.49	2.57 ± 0.39	N.A.	10.59 ± 0.97	10.81 ± 0.15
	-0.70	300	49.38 ± 0.51	23.05 ± 1.08	2.59 ± 0.51	1.84 ± 0.82	0.02 ± 0.04	7.37 ± 1.37	11.87 ± 1.91
	-0.74	400	47.6 ± 0.44	21.32 ± 0.75	2.42 ± 0.75	1.71 ± 0.68	0.50 ± 0.15	7.26 ± 0.94	16.41 ± 1.19
	-0.78	500	41.67 ± 0.45	18.42 ± 0.65	1.79 ± 0.63	1.41 ± 0.64	1.67 ± 0.63	7.7 ± 0.84	24.33 ± 1.15
	-0.82	600	36.79 ± 0.36	16.47 ± 1.64	1.26 ± 0.55	0.78 ± 0.21	3.81 ± 0.04	7.9 ± 0.76	30.57 ± 1.28
Ag/Cu	-0.58	100	41.39 ± 1.71	14.76± 0.49	5.29 ± 1.54	3.88 ± 0.14	N.A.	14.28 ± 0.92	15.23 ± 2.99
	-0.64	200	47.49 ± 0.23	22.27 ± 1.29	3.66 ± 0.71	2.52 ± 0.48	N.A.	10.32 ± 1.02	9.74 ± 0.16
	-0.68	300	49.15 ± 0.87	23.74 ± 0.53	3.13 ± 0.50	1.89 ± 0.75	0.05 ± 0.08	8.14 ± 0.26	10.34 ± 1.92
	-0.72	400	46.92 ± 1.72	22.82 ± 0.20	2.94 ± 0.02	1.73 ± 0.65	0.38 ± 0.19	8.49 ± 1.49	14.02 ± 1.20
	-0.75	500	39.57 ± 1.26	17.18 ± 1.54	2.06 ± 0.44	1.44 ± 0.61	3.21 ± 1.49	8.31 ± 1.35	23.66 ± 1.16
	-0.78	600	33.34 ± 2.75	15.34 ± 0.59	1.30 ± 0.53	0.79 ± 0.21	5.02 ± 1.46	7.47 ± 0.88	30.08 ± 3.84
DDT-Ag/Cu	-0.60	100	18.82 ± 1.14	18.09 ± 0.92	5.07 ± 0.05	4.62 ± 1.16	0.31 ± 0.27	34.09 ± 3.48	8.90 ± 1.56
	-0.69	200	28.11 ± 1.16	29.30 ± 1.18	4.23 ± 0.48	4.70 ± 0.41	0.66 ± 0.57	20.2 ± 0.1	9.29 ± 1.25
	-0.74	300	33.13 ± 1.16	31.27 ± 0.69	3.76 ± 0.70	4.11 ± 0.54	0.99 ± 0.77	15.49 ± 1.41	8.02 ± 1.43

	-0.77	400	34.89 ± 0.31	34.11 ± 1.41	3.52 ± 0.39	2.88 ± 0.62	1.49 ± 0.99	9.73 ± 1.08	8.24 ± 0.51
	-0.80	500	35.91 ± 0.76	34.84 ± 1.29	3.33 ± 0.42	2.77 ± 0.62	1.06 ± 0.39	7.4 ± 0.65	8.64 ± 0.14
	-0.82	600	32.3 ± 1.03	28.16 ± 2.52	3.28 ± 0.32	2.43 ± 0.57	1.93 ± 0.53	7.51 ± 0.02	8.64 ± 0.68
